# WildSpan: mining structured motifs from protein sequences

**DOI:** 10.1186/1748-7188-6-6

**Published:** 2011-03-31

**Authors:** Chen-Ming Hsu, Chien-Yu Chen, Baw-Jhiune Liu

**Affiliations:** 1 Department of Computer Science and Information Engineering, Ching Yun University, Jung-Li, 320, Taiwan; 2 Department of Bio-Industrial Mechatronics Engineering, National Taiwan University, Taipei, 106, Taiwan; 3 Department of Computer Science and Engineering, Yuan Ze University, Jung-Li, 320, Taiwan

## Abstract

**Background:**

Automatic extraction of motifs from biological sequences is an important research problem in study of molecular biology. For proteins, it is desired to discover sequence motifs containing a large number of wildcard symbols, as the residues associated with functional sites are usually largely separated in sequences. Discovering such patterns is time-consuming because abundant combinations exist when long gaps (a gap consists of one or more successive wildcards) are considered. Mining algorithms often employ constraints to narrow down the search space in order to increase efficiency. However, improper constraint models might degrade the sensitivity and specificity of the motifs discovered by computational methods. We previously proposed a new constraint model to handle large wildcard regions for discovering functional motifs of proteins. The patterns that satisfy the proposed constraint model are called W-patterns. A W-pattern is a structured motif that groups motif symbols into pattern blocks interleaved with large irregular gaps. Considering large gaps reflects the fact that functional residues are not always from a single region of protein sequences, and restricting motif symbols into clusters corresponds to the observation that short motifs are frequently present within protein families. To efficiently discover W-patterns for large-scale sequence annotation and function prediction, this paper first formally introduces the problem to solve and proposes an algorithm named WildSpan (sequential pattern mining across large wildcard regions) that incorporates several pruning strategies to largely reduce the mining cost.

**Results:**

WildSpan is shown to efficiently find W-patterns containing conserved residues that are far separated in sequences. We conducted experiments with two mining strategies, protein-based and family-based mining, to evaluate the usefulness of W-patterns and performance of WildSpan. The protein-based mining mode of WildSpan is developed for discovering functional regions of a single protein by referring to a set of related sequences (e.g. its homologues). The discovered W-patterns are used to characterize the protein sequence and the results are compared with the conserved positions identified by multiple sequence alignment (MSA). The family-based mining mode of WildSpan is developed for extracting sequence signatures for a group of related proteins (e.g. a protein family) for protein function classification. In this situation, the discovered W-patterns are compared with PROSITE patterns as well as the patterns generated by three existing methods performing the similar task. Finally, analysis on execution time of running WildSpan reveals that the proposed pruning strategy is effective in improving the scalability of the proposed algorithm.

**Conclusions:**

The mining results conducted in this study reveal that WildSpan is efficient and effective in discovering functional signatures of proteins directly from sequences. The proposed pruning strategy is effective in improving the scalability of WildSpan. It is demonstrated in this study that the W-patterns discovered by WildSpan provides useful information in characterizing protein sequences. The WildSpan executable and open source codes are available on the web (http://biominer.csie.cyu.edu.tw/wildspan).

## Background

As sequencing projects generate biological sequences at an astonishing rate, identifying functional signatures directly from sequences is of particular value in functional biology [1,2]. These signatures can then be used to predict function or functionally important residues of a novel protein. The functionally important residues of proteins are generally conserved during evolution [3]. Conserved regions of a protein sequence can be identified by aligning the query protein with its homologues in protein databases. Alternatively, pattern mining (also called motif discovery) is an effective approach for identifying conserved regions [4-7].

Motif finding algorithms have been widely used in this field for finding sequence signatures when given a set of related sequences (pattern mining). The resultant motifs are then employed in predicting protein function and functional sites when given a novel sequence (pattern matching). We previously employed motif finding in a hybrid way: detecting functional regions of a novel sequence directly by mining its sequence along with a set of homologues found in sequence database (MAGIIC-PRO, [8]). Similar to multiple sequence alignment (MSA), MAGIIC-PRO can be invoked as long as the query protein can find sufficient homologues from databases (this can be easily achieved after the completion of abundant sequencing projects). In this way, functional residues of the query protein can be predicted even when the function of the collected homologues is still left unknown. MAGIIC-PRO identified a set of residues that are concurrently conserved during evolution. This can supplement the conservation information provided by MSA.

PROSITE language is one of the formal ways to express a pattern [9]. A capital letter in a pattern is called an exact symbol. For example, the pattern 'K-x-L-x(2)-E-x(2,3)-G' have four exact symbols. In addition to capital letters, a pattern also contains wildcards, expressed by the symbol 'x'. A wildcard can match any letters in a biological sequence. This pattern matches any sequence containing a substring which starts with 'K', followed by an arbitrary letter, followed by 'L', followed by two arbitrary letters, followed by 'E', followed by two to three arbitrary letters, and ends with 'G'. Both 'x' and 'x(2)' are called rigid gaps, a gap of fixed length. A rigid gap can match a certain number of successive residues on which mutations are allowed. On the other hand, x(2,3) is a flexible gap, a gap of irregular length. A flexible gap can match a number of residues on which not only mutations are present but also insertions or deletions are allowed.

**Figure 1:**
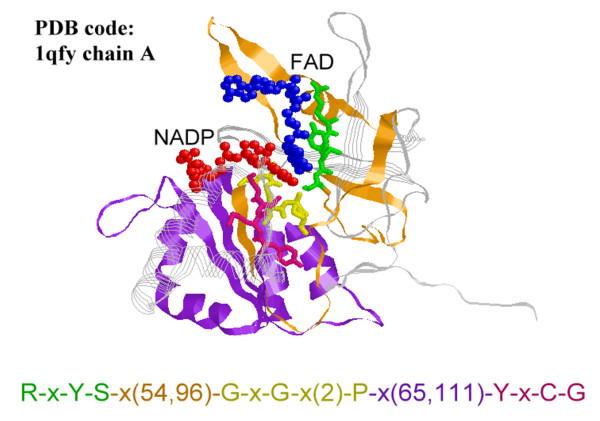
**An example of structued motifs **This motif is observed on the protein Ferredoxin-NADP reductase [Swiss-Prot: P10933] and additional 150 Oxidoreductase FAD/NAD(P)-binding proteins from the InterPro entry [InterPro: IPR001433]. The motif is consisted of three local conserved regions 'R-x-Y-S', 'G-x-G-x(2)-P', and 'Y-x-C-G', interleaved by two large gaps x(54,96) and x(65,111). When these three pattern blocks are mapped onto the 3D structure of Ferredoxin-NADP reductase, it is shown that all the three blocks are close to the FAD/NAD(P) binding site. Pattern blocks are plotted in *sticks *using different colors. The long gap between the first and the second blocks (the second and the third blocks) is plotted with *ribbons *in orange (purple). The ligands FAD and NADP are shown as *ball-and-stick *in blue and red, respectively.

For proteins, the residues associated with a functional site are not necessarily found in a local region of the sequence [5,7,10,11]. Rather, the residues of a functional site are commonly clustered into several local regions that together constitute an important substructure when the protein is folded. It is observed that within protein families, only limited flexibility is allowed in such local conserved regions, while large irregular gaps may be present in between these regions as long as the inserted or deleted segments do not affect the functionality of the proteins [3,12-14]. In Figure 1, we provide an example of such structured motifs. A structured motif 'R-x-Y-S-x(54,96)-G-x-G-x(2)-P-x(65,111)-Y-x-C-G' is observed on the protein Ferredoxin-NADP [Swiss-Prot accession number: P10933] and additional 150 Oxidoreductase FAD/NAD(P)-binding proteins belonging to the same protein family [InterPro entry: IPR001433] with P10933. This motif contains three blocks, and two inter-block gaps, 'x(54,96)' and 'x(65,111)', are quite large and flexible. It is shown in Figure 1 that the three pattern blocks, though largely apart in sequence, are clustered together in three-dimensional space and corporately form a binding region associated with the binding of flavin adenine dinucleotide (FAD) and nicotinamide adenine dinucleotide phosphate (NADP) ligands. This observation motivates the current study to develop an algorithm for discovering sequence motifs that contain large flexible gaps in between the clusters of exact symbols. Though such structured motifs have been introduced and analyzed in studies related to cis-regulatory elements in DNA [15-18], few algorithms have been particularly designed for protein sequence analysis [15,19].

Discovering functional signatures with large irregular gaps complicates mining procedures. Motif finding algorithms typically use constraints to produce specific types of patterns expected by the users. Table 1 summarizes several well-known constraint models for handling gaps when conducting motif finding in biological sequences. Algorithms that consider only short conserved words (without gaps) [5,20] or rigid gaps [4,6,21-23] efficiently and effectively identify short motifs (model 1). However, such models impose limitations on the search space of the patterns that can be discovered because no insertions or deletions are allowed across sequences. On the other hand, Pratt algorithm [19] introduces the concept of gap flexibility to enlarge the search space (model 2). A more general type of constraint models sets the lower and upper bound of a gap respectively (model 3). However, allowing large flexible gaps in between any two adjacent exact symbols induces noisy patterns and also worsens system performance [24]. Another gap constraint model considers a set of continuous words that are interleaved with unlimited flexible gaps (model 4) [7,11,14]. This model is valuable since the large insertions and deletions that occur during evolution can be properly handled. However, employing continuous words for locally conserved regions limits their application in the analysis of protein sequences, in which conservative substitutions are frequently observed. In addition, the unlimited gap flexibility in model 4 also results in noises.

**Table 1:** Constraint models of gapped motifs employed in previous studies

Gap constraint models	Descriptions	Examples of existing algorithms
**Model 1**	At least L non-wildcards should be present in a pattern of maximum length of W. (e.g. 'A-x-K-H-x(2)- E')	Teiresias [6] and SPLASH [4]

**Model 2**	A gap with a maximum flexibility FL is allowed between any pair of pattern symbols; related constraints: maximum number of flexible gaps, maximum product of each flexibility. (e.g. 'A-x(2,3)-W-x-H-(4,6)-E')	Pratt [19]

**Model 3**	A gap with a minimum length of LB (e.g. LB = 1) and a maximum length UB (e.g. UB = 10) is allowed in between any pair of pattern symbols. (e.g. 'A-W-x(1,5)-H-x(4,10)-E')	Ref. [35,36]

**Model 4**	A gap of any length (denoted as *) is allowed in between any pair of continuous words in a pattern; related constraints: minimum length of continuous words. (e.g. 'A-W-D-A-x(*)-H-E-D-x(*)-K-R')	Ref. [7,11,14]

**Model 5**	a gap with a minimum length of LB and a maximum length of UB is allowed in between any pair of symbols in a pattern block; a gap with a minimum length of LB" and a maximum length of UB" is allowed in between any pair of pattern blocks; related constraints: minimum length of pattern block; (e.g. MAGIIC [24]:'A-W-x(2,3)-H-x(45, 60)-E-x-D-x(1,2)-K', a pattern block is underscored), RISOTTO [15] (e.g. R-G-I-T-I-T-x(16,18)-P-G-H-A-D-F, one mismatch is allowed in a pattern block).	MAGIIC [24] and RISOTTO [15]

The model 5 presented in Table 1 was previously proposed in our recent work [24] The algorithm MAGIIC utilizes a combination of intra- and inter-block gap constraints to discover structured motifs like 'A-x-C-x(2,3)-D-F-x(10,198)-R-G-x(0,1)-D'. Such patterns have its symbols clustered into many pattern blocks, where the gaps within a pattern block are called intra-block gaps and the gaps between two successive blocks are called inter-block gaps. We have demonstrated in the previous study [24] that using the combination of intra- and inter-block gap constraints greatly improves mining efficiency. The MAGIIC patterns are similar to the structured motifs proposed for discovering cis-regulatory elements [15]. Though initially developed for mining DNA sequences, the package RISOTTO can also be used for mining protein sequences.

After largely using MAGIIC to identify functional motifs of protein sequences, we observed that restricting intra-block gaps to only rigid gaps can further refine the mining results greatly. In this regard, the later proposed web server MAGIIC-PRO simply employs rigid intra-block gaps to handle local mutations. In MAGIIC-PRO, the maximum length of a rigid intra-block gap is set to a small value, such as two or three. Regarding the inter-block gaps, both MAGIIC and RISOTTO set the minimum (a lower bound) and maximum (an upper bound) distances between blocks in advance. When developing MAGIIC-PRO, we observed that setting the minimum and maximum distances between blocks prior to motif discovery is very difficult. This problem can be resolved when a query protein is involved during pattern mining. That is, the minimum and maximum distances between blocks can be set dynamically according to the gaps present in the query sequence. With the length of the gaps observed in the query sequence, a novel constraint named 'maximum relative flexibility' was designed to calculate the lower and upper bounds that are allowed among the homologues for this particular gap. Patterns satisfying the constraint model proposed in MAGIIC-PRO are called W-patterns.

This study aims at introducing the algorithm WildSpan for efficiently discovering W-patterns. In this paper, we demonstrated that the constraint 'maximum relative flexibility' has some good properties, and thus aggressive pruning strategies can be employed by WildSpan to improve efficiency. The performance of WildSpan is evaluated in two ways. Comparison of W-patterns to annotated motifs in existing databases reveals that W-patterns can capture the functional signatures of proteins well. Comparison of WildSpan to existing algorithms that perform the similar task reveals that W-patterns are more powerful in detecting protein functional regions than currently existing constraint models.

In this paper, we also illustrate how WildSpan can be invoked as the protein-based or family-based mining mode for future proteomics applications. The mining results of protein-based mining reveal that WildSpan can efficiently and effectively identify functional or structural signatures of the query protein directly from the protein sequences. On the other hand, the mining results of family-based mining reveal that WildSpan can be used to identify sequence signatures of protein families for future function prediction and sequence annotation. The idea of protein-based mining has been integrated in our web servers MAGIIC-PRO [8] in 2006 and iPDA [25] in 2007 for annotating protein sequences. On the other hand, the idea of family-based mining has been integrated in the web server E1DS in 2008 [26] for predicting enzyme catalytic sites and residues. In summary, though several independent studies have successfully shown the usefulness of the constraint model W-patterns, the design of the WildSpan algorithm has not been previously addressed and published elsewhere. In addition, the standalone package and open source codes of WildSpan are now ready for downloading and can be used for large-scale proteome studies in the future.

## Results and Discussion

This section evaluates the efficiency and effectiveness of WildSpan in identifying functional regions of protein sequences. First, we conduct experiments on a protein-protein docking benchmark [27] for evaluating the performance of the protein-based mining mode of WildSpan in identifying functionally important regions of proteins. By this dataset we demonstrate that WildSpan is capable of identifying sequence motifs that usually contribute to forming local structures of proteins and are related to functional interfaces. Next, we execute WildSpan in family-based mining mode, and investigate the potential of the W-patterns to serve as diagnostic patterns for a protein family. After that, we investigate the effect of algorithm parameters on the mining results, and finally the scalability of WildSpan is evaluated using datasets containing different numbers of input sequences as well as with different maximum lengths. All the experiments are conducted on a 3.4 GHz Intel PC machine with 2 GB main memory, running Linux Fedora 9 operating system.

### Experiments on detection of protein functional regions

The protein-based mining mode of WildSpan aims at discovering functional regions for a query protein based on a set of homologues found in sequence databases. The performance of WildSpan in this task is evaluated from two aspects: (a) whether the blocks separated in sequence cluster together in three-dimensional protein structure; and (b) whether the conservation information provided by W-patterns is more function-related than that derived from MSA. On the other hand, the family-based mining mode of WildSpan aims at deriving motifs that characterize the functional signatures of a given family. The performance of WildSpan in this task is evaluated by investigating the accuracy of function classification by using W-patterns, compared with the curated patterns provided in PROSITE and the patterns discovered by three existing motif finding packages.

#### Protein-based mining

For protein-based mining, it has been demonstrated in our previous study [28] that the W-patterns can be used to facilitate identifying the binding interface of protein-protein complexes. Here, we repeated the same evaluation procedure by using the same benchmark, the protein-protein docking benchmark 2.0 established by the ZDOCK team [27], but recollect the homologue set for each query protein from a newer version of sequence databases (Oct. 10, 2008).

The complete procedures for identifying interacting interfaces for a query protein are as follows:

(1) For a query protein chain, the input data (homologues of the query, 150 at most) fed to WildSpan was obtained by performing PSI-BLAST [29] against Swiss-Prot database [30] using BLOSUM62 substitution matrix and an E-value cut-off of 0.01. The sequences nearly identical to the query protein (sequence identity > 90%) or with a low identity (sequence identity < 30%) were excluded from the input data. If the homologues of query protein are not sufficient in Swiss-Prot database (< 5 homologues), the process of collecting homologues was executed one more time against the non-redundant (NR) database [29].

(2) Invoking WildSpan for pattern mining: at least one W-pattern with five blocks is discovered for each query protein. Different settings regarding the number of blocks in a W-pattern have been tested from two to six, while the setting 'five' achieved the best performance (data not shown). The maximum relative flexibility is set as 50%. Other parameter settings remain as default. The discussions regarding how the default settings of WildSpan were determined can be found in Additional file 1. Like other motif finding algorithms, it is challenging to have all the parameters set to proper values in a single run of WildSpan. A loose setting of parameters results in too many patterns that confuse the users, while a tight setting results in no patterns at all. To achieve the goal of delivering a five-block W-pattern with a support as high as possible for each query protein, we follow a procedure of automated parameter tuning when invoking WildSpan. A flowchart illustrating how WildSpan was invoked with different parameter settings to complete the mining task was provided in Figure A1.2 of Additional file 1.

(3) In the end of motif finding, a consensus motif that merges all the discovered W-patterns is examined for evaluating the mining results for each query protein.

**Figure 2:**
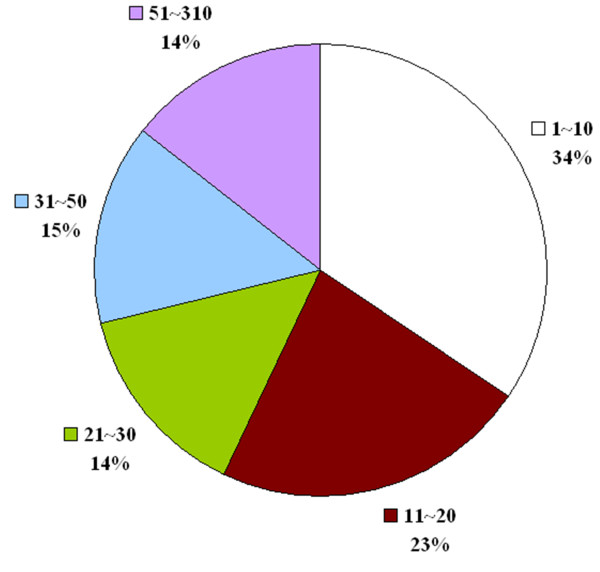
**Distribution of inter-block gap length observed among the query proteins of the protein-protein docking benchmark**.

Among the 220 protein chains in this benchmark, 217 protein chains can find sufficient (≥5) homologues for motif discovery. For all the 217 query proteins, WildSpan successfully found at least one motif containing five blocks. There are in total 1011 motif blocks discovered by WildSpan. Each block contains 10 residues in average, including positions that allow for mutations. In Figure 2, the distribution of the length of inter-block gaps observed on the 217 query proteins is provided. More than one-fourth (29%) of the inter-block gap have a length longer than 30 residues. Though these blocks are interleaved with long gaps in sequence, it is shown in Table 2 that the conserved blocks in W-patterns usually cluster together in space (92.7% of the discovered pattern blocks contains an atom that is within 5Å to an atom of another block belonging to the same W-pattern). This proportion is significantly higher than that of a randomly generated motif (80.1%) containing five blocks, which each contains 10 residues.

**Table 2:** Comparison of W-patterns with randomly generated patterns

	Number of predicted blocks	Number of blocks in average	Length of predicted blocks in average	Cluster propensity
**W-patterns**	1011	4.7	10	92.7%

**Randomly generated patterns (average of 10 rounds)**	1041	4.8	9.4	80.1%

The clustering propensity of W-patterns generated by WildSpan was compared with randomly generated patterns. The experiments were tested on the 217 of 220 protein chains (PP220) in the protein-protein benchmark (no homologues can be found for the three cases: 1ml0_A, 1ml0_B, 1udi_I).

The results above reveal that some of the residues in W-patterns might be conserved for structure conformation. The next question to answer is whether the residues in W-patterns are conserved for function conservation. In this regard, we further evaluate the quality of a W-pattern by calculating the proportion of interface residues in a W-pattern. It is shown in Table 3 that 23.6% of the residues in the W-patterns are close to the binding partner in protein-protein complexes within 5Å. Since MSA is widely adopted to discover conserved residues for the query protein with respect to its homologues, the conserved residues detected by techniques based on MSA were compared here. To compare with MSA, we calculated the conservation scores based on the alignment of Clustal-W using the iPDA web server. In Table 3, it is shown that only 18.7% of the conserved residues detected by MSA are interface residues. This reveals that WildSpan is able to discover more conserved residues that are related to protein function.

**Table 3:** Comparison of the conservation information provided by WildSpan with that of MSA

	Total number of residues characterized as conserved	Number of interface residues in the group of residues categorized as conserved	Proportion of interface residues in the group of residues categorized as conserved
**W-patterns**	10268	2351	23.6%

**MSA**	10638	2058	18.7%

We investigated the property of W-patterns at residue level by calculating the proportion of interface residues in W-patterns. The results are compared with the conserved residues assigned by Clustal-W (MSA). The experiments were tested on the 217 of 220 protein chains (PP220) in the protein-protein benchmark (no homologues can be found for the three cases: 1ml0_A, 1ml0_B, 1udi_I).

#### Family-based mining

In this experiment, we show the potential of the W-patterns found by invoking the family-based mining mode of WildSpan to serve as the diagnostic patterns for protein families. Instead of using only one pattern as the classification rule, we propose using multiple patterns as the discriminator. The PROSITE database contains diagnostic patterns for protein families, domains, and functional sites. The ten largest PROSITE groups are collected as the training data (PA10F), and the W-patterns found by the family-based mining mode of WildSpan will be compared with the PROSITE patterns of that input set. It is nominally required that each pattern contains at least three pattern blocks, but patterns containing nine or more exact symbols though only belonging to one or two blocks will also be reported and selected. When these ten PROSITE families were analyzed using WildSpan, the maximum relative flexibility of an inter-block gap is set as *f*_max _= 50% and the other parameters are set as default.

The protein sequences of each family in PA10F were collected based on the functional annotation in an earlier release of Swiss-Prot database as shown in Table A2.1 of Additional file 2. Meanwhile, all the protein sequences collected from a recent release of Swiss-Prot database were adopted as the testing data. A sequence is categorized as a positive sample as long as it matches any of the patterns derived by WildSpan. The sensitivity (TP/(TP+FN)), precision (TP/(TP+FP)) and specificity (TN/(TN+FP)) of the selected patterns are compared with those of the diagnostic pattern from the PROSITE database, where TP, FP, TN, and FN denote the number of true positives, false positives, true negatives, and false negatives, respectively. It should be noted that the training and testing procedures adopted here are not like a standard machine learning approach in two ways. First, no negative samples are involved in the training procedure. With the positive sequences only, motif finding algorithms are expected to achieve the maximum sensitivity rate over the input set under the user-specified constraints. Second, most of the training samples are included in the testing data as well. In this regard, it is expected that the sensitivity rates should be high, but obviously not all the methods fulfil this expectation. Another focus will be on how good the specificity rates can be achieved by different methods.

**Figure 3:**
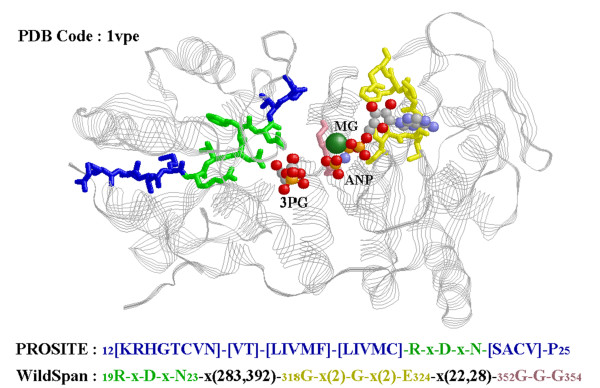
**A W-pattern versus the PROSITE pattern for a family of interest**. The W-pattern derived by WildSpan for Phosphoglycerate kinase (PS00111) versus the PROSITE pattern. The small numbers in patterns are the residues IDs in the PDB structure.

Table 4 reveals that W-pattern is good in characterizing new proteins (eliminating false positives while keeping satisfied sensitivity rates). The predictions are compared to PROSITE patterns and the motifs discovered by motif-finding algorithms, RISOTTO, Pratt, and Teiresias. While providing a competitive predicting ability when compared to the PROSITE patterns, we observed that the W-patterns derived by WildSpan provide more complete and precise signatures regarding the binding regions than the PROSITE patterns, as exemplified in Figure 3. Complete results for protein function classification are shown in Table A2.2 of Additional file 2. It is concluded that W-patterns perform similarly to the curated patterns in PROSITE and outperforms the motifs discovered by the other three constraint models.

**Table 4:** Experimental results for protein family classification

Method/Database	Time used in seconds	Sensitivity	Precision	Specificity	MCC^1^
**PROSITE**	-	85.717	93.043	99.996	0.857
**RISOTTO**	18.635	47.003	99.957	100	0.470
**Pratt**	1598.3	81.507	94.159	99.995	0.815
**Teiresias**	0.908	76.798	0.2523	41.163	0.030
**WildSpan (Family-based)**	89.782	99.042	97.481	99.993	0.990

The table shows the performance of family-based mining of WildSpan on protein family classification based on PA10F. The results were compared to PROSITE annotated patterns and three other pattern mining methods: RISOTTO, Teiresias, and Pratt. The input data was prepared by collecting proteins in the release 50.9 of UniProtKB/Swiss-Prot (235673 entries), and the discovered patterns were verified through all protein sequences in the release 2010/08 of UniProtKB/Swiss-Port (518415 entries). Fragment and partially matches were excluded in both training and testing data. The parameter values of all the methods were set as default

^1 ^Matthews correlation coefficient (MCC): (*TP*×*TN - FP*×*FN*)/SQRT( (*TP*+*FP*) × (*TP*+*FN*) × (*FN*+*FP*) × (*TN*+*FN*) )

We observed that the false positives reported in Table 4 are not really wrong predictions. For example, most of proteins are annotated in another database (i.e. Pfam) as the target function. In Table 5, we provided the details about the number of false positives that can actually find annotation from another database. These results show the potential of the W-patterns in predicting protein functions with both high sensitivity and specificity. This also explains why the E1DS server [26] performs well in predicting catalytic sites and residues when invoking the family-based mining mode of WildSpan to construct the signature database.

**Table 5:** Many false positives of WildSpan are not really false positives

PROSITE family	False positives (FPs)/the number of FPs that actually are annotated as the target function by other databases				
	
	WildSpan (Family-based)	PROSITE	RISOTTO	Pratt	Teiresias
**PS00301**	196/196	0/0	1/1	8/5	341227/NA
**PS00469**	1/1	6/0	0/0	0/0	0/0
**PS00455**	115/6	23/0	0/0	0/0	350060/NA
**PS00111**	1/1	0/0	2/1	10/1	263012/NA
**PS00113**	4/2	0/0	0/0	109/7	0/0
**PS01071**	0/0	2/0	0/0	0/0	380979/NA
**PS00627**	17/4	3/0	0/0	0/0	381040/NA
**PS00387**	0/0	102/0	0/0	0/0	31339/NA
**PS00112**	0/0	0/0	0/0	0/0	31339/NA
**PS00485**	1/1	20/0	0/0	150/0	350533/NA

NA: information not available because the number of false positives is too large to manually validate the protein function.

### Performance analysis

In this section, we investigate the efficiency of WildSpan in identifying W-patterns based on the ten datasets in PA10F.

#### Performance study on pattern pruning

To evaluate the efficiency of WildSpan with the proposed pruning strategy, we evaluated the performance of two versions of WildSpan algorithm as follows.

(a) WildSpan: the WildSpan algorithm with pruning strategies in the second phase.

(b) WildSpan-NP: the WildSpan algorithm with exhaustive search in the second phase by enumerating all combinations.

**Figure 4:**
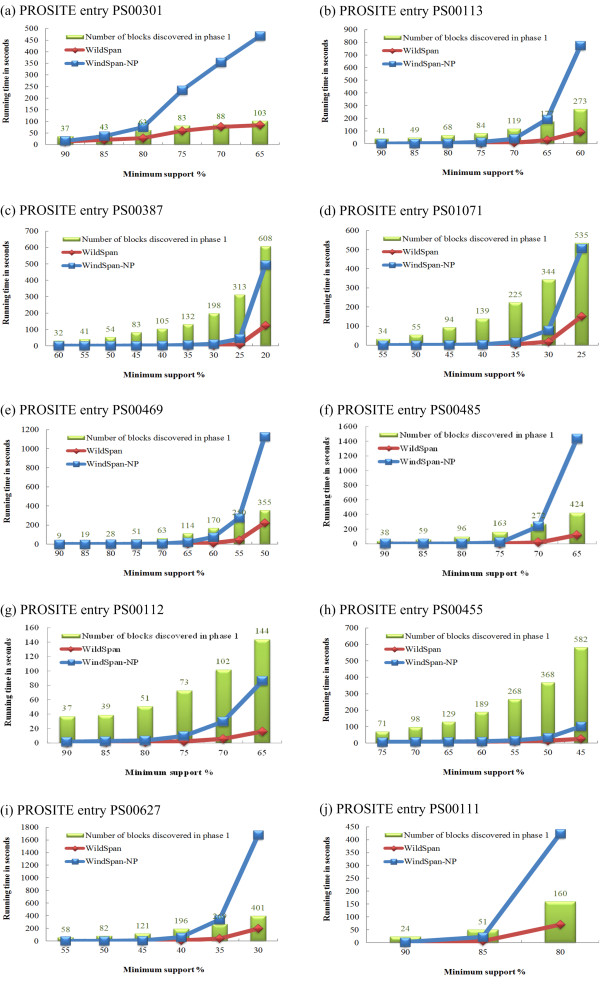
**Performance comparison**. This figure shows the running time of WildSpan versus WildSpan with no pruning (WildSpan-NP) on the PA10F dataset.

The experimental results on PA10F with different minimum support thresholds are shown in Figure 4. For each dataset, the other parameters were set as: *κ*_min _= 3, *γ*_max _= 3, *n*_min_= 2, and *f*_max _= 50%, which denote the minimum size of a block, the maximum length of an intra-block gap, the minimum number of blocks in a W-pattern, and the relative flexibility constraint, respectively. As depicted in the Figure 4, WildSpan is in several orders of magnitude faster than WildSpan-NP for all the cases. When the support threshold is high, the performance curves of WildSpan and WildSpan-NP are close. This is because fewer candidates of blocks exist for higher values of minimum support. However, WildSpan with lower supports achieves a better reduction in terms of search space and consequently provides a better speedup, since there are many candidate blocks and WildSpan-NP enumerates all the combinations, which is computationally expensive.

**Figure 5:**
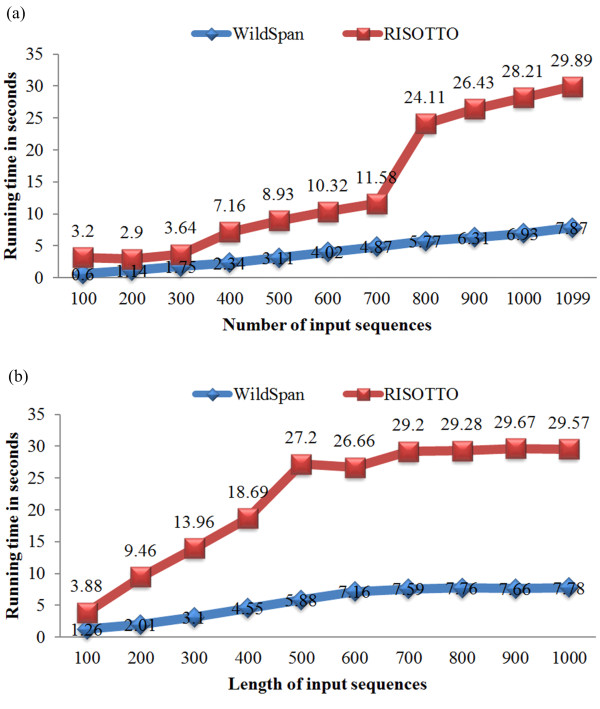
**Study of scalibility of WildSpan**. Study on the effect of varying the number and sequence length of input sequences of input sequences fed to WildSpan based on the largest dataset (PS00301) of PA10F. (a) Analysis of varying the number. (b) Analysis of varying length of input sequences fed to WildSpan.

On the other hand, the scalability of WildSpan is investigated by studying the effect of varying length and input size of input datasets. The employed dataset is the largest family PS00301 of PA10F, which contains 1099 protein sequence members, {*s*_1_, *s*_2_,..., *s*_1099_}. We randomly selected *x *proteins from PS00301 as the input data, *x *∈ {100, 200, ..., 1000, 1099}. These eleven input sets were used to test the scalability of WildSpan versus the number of input sequences. Figure 5(a) shows the analysis, and the scalability of WildSpan is compared with RISOTTO. We also generated another test sets in which the maximum length *y *of input sequences is restricted, *y *∈ {100, 200, ..., 1000}. These ten input sets were used to test the scalability of WildSpan when the length of input sequences is increasing. Again, the result was compared with RISOTTO, as shown in Figure 5(b). For both RISOTTO and WildSpan, the minimum support threshold is set as a proper value such that a pattern with a support as high as possible can be found. We have validated that all of W-patterns with the maximum support are directly associated with the functional sites of the query protein by examining locations of the discovered patterns on available protein structures.

## Conclusions

This paper presents an algorithm WildSpan for discovering W-patterns. Discovering W-patterns is important in analyzing protein sequences because protein functional motifs are usually composed of many conserved blocks that are separated in primary sequences but are often close to each other in 3-D structures. The constraint model (W-patterns) and the developed mining and pruning strategies (incorporated in WildSpan) is shown to efficiently and effectively deliver information concerning co-occurred sequence conservation. The derived W-patterns was previously shown to be useful in predicting intra-molecular interactions, identifying hot regions of protein-protein complexes, and detecting binding regions of protein-ligand interactions [8,31-33]. To facilitate using the proposed algorithm in future application, we implemented a stand-alone program and provided a user-friendly web server for WildSpan to help the biological community in discovering functional regions of protein sequences in a large scale. WildSpan was developed using C/C++ with the support of C++ Standard Template Library under Linux, and has been tested on various GNU/Linux platforms, including Red Hat 9.0 and Fedora 5 or higher. It should also work well with other UNIX-like operating systems.

## Methods

This work introduces a two-phase algorithm (called WildSpan) to efficiently discover W-patterns when given a query sequence along with a set of homologous sequences. In the first phase, WildSpan constructs the complete set of blocks with rigid-length gaps using a bounded-gap prefix-growth approach. In the second phase, WildSpan discovers W-patterns by connecting any pairs of candidate blocks with large flexible gaps. Several pruning strategies are employed in the mining process to improve the performance. This section first formally describes the problem statement and the associated terminology. After that, the algorithm WildSpan is described step by step.

### Problem Statement

Given a query sequence *S_q_*, a sequence database *D*, and a parameter set *θ *regarding pattern block, W-pattern and gap constraints, the algorithm will find the complete set of closed W-patterns (the definition of closed patterns is provided in Additional file 3) present in the sequence database *D *such that each W-pattern satisfies the constraints in *θ *and its matched sequences include the query sequence *S_q_*. The parameter set *θ *includes the minimum support (minimum occurrences) of the W-pattern, the minimum number of blocks in a W-pattern, the minimum number of exact symbols in a block, the maximum length of an intra-block gap between two adjacent exact symbols in a block, and the maximum flexibility of an inter-block gap between two adjacent blocks in a W-pattern.

A block or W-pattern is called 'satisfied' if it agrees with all the user-specified constraints. Each constraint will be defined when it is first used in the description of the algorithm.

#### Definition 1. (Sequence and sequence database)

A sequence over an alphabet *Σ *is a finite sequence of symbols belonging to *Σ*, e.g., protein sequence is sequence over a 20-letter alphabet. For any sequence *S*=〈*a*_1_...*a_m_*〉, a sequence *S_x _*is called a subsequence of *S*, if *S_x _*can be obtained by deleting zero or more symbols from sequence *S*. We use *S*[*i*..*j*] to denote the substring 〈*a_i_*...*a_j_*〉 (contiguous subsequence) of *S*, which starts at position *i *and ends at position *j *of *S*, for 1 ≤ *i *≤ *j *≤ *m*. In particular, *S*[1..*i*] is the prefix of sequence *S *that ends at position *i*, and *S*[*i*..*m*] is the suffix of sequence *S *that begins at position *i*. The length of sequence *S*, denoted as *m*, is defined as the number of symbols in *S*. An input sequence database *D *contains a set of sequences.

In general, the input sequence database is a set of protein sequences that are presumed to be functionally or evolutionarily related to the query protein (the first sequence in *D*). Patterns found in protein sequences can be expressed in PROSITE language. For our purpose we need a more formal definition as below.

#### Definition 2. (Pattern)

A pattern *P *can be written as *P *= *a*_1_-x(*i*_1_, *j*_1_)-*a*_2_- x(*i*_2_, *j*_2_)-...-x(*i_p-1_*,*j_p-1_*)-*a_p _*in PROSITE language, where *a*_1_,...,*a_p _*are the exact symbols of *P*, and x(*i_x_*, *j_x_*) are the wildcard regions (i.e. gaps) of *P *for *i_x _*≤ *j_x _*(1 ≤ *x *<*p*). A pattern *Q *is a sub-pattern of *P *if *Q *can be obtained by deleting one or more exact symbol(s) from *P*. Conversely, *P *is a super-pattern of *Q*. We say that a sequence *S *matches the pattern *P *if *S *contains a substring that can be derived from *P *by substituting each wildcard symbol 'x' by an arbitrary symbol from *Σ*. The set *S*/*P *stands for all the substrings of *S *that match pattern *P*. The notation x(*n*,*m*), 0 ≤ *n *<*m*, is used for a wildcard region with minimum length gap of *n *and maximum length gap of *m*, and x(*n*) stands for a rigid-length *n *gap. The wildcard "-x(*n*)-" is simplified as "-" if *n *= 0, and is represented as x if *n *= 1.

The first constraint of the algorithm WildSpan is the minimum support constraint *λ*.

#### Definition 3. (Minimum support constraint)

The support of a satisfied pattern *P *(block or W-pattern) is defined as the percentage of the distinct input sequences *S *∈ *D *such that *S *matches *P *under the constraints in *θ*. Such matched sequences are called supporting sequences of *P*. On the other hand, the non-matched sequences of *P *in *D *are called excluded sequences of *P*. A pattern *P *will be reported if and only if its support is greater than or equal to the minimum support constraint *λ *and satisfy all constraints in *θ*.

The minimum support constraint is critical to the quality of mining results, but it is difficult to determine in advance since the minimum support of satisfied patterns cannot be accessed before they are discovered. A lower value on this constraint yields more patterns. In this regard, this parameter can be set in the following way: WildSpan begins with a large support, e.g. 100%, and decreases this setting gradually until a desired number of satisfied patterns have been found.

##### Phase 1: identifying rigid-gapped blocks

The first phase of the WildSpan algorithm finds all of the closed blocks with a support >*λ *and which satisfy the constraints concerning a block. The definitions of a block and related constraints are as follows.

#### Definition 4. (Block and intra-block gap)

A block (short for pattern block) *Ψ *= *a*_1_-x(*i*_1_)-*a*_2_-x(*i*_2_)-...-x(*i_b-1_*)-*a_b _*is a short pattern in which only rigid-length gaps are allowed. The size of a block is defined as the number of exact symbols inside it. The gap between any two adjacent symbols within a block is called an *intra-block gap*. The maximum length of an intra-block gap is set by the constraint *γ*_max _and the minimum size of a block is specified by the constraint *κ*_min_.

To grow a block from scratch when gaps are considered, we invoke a procedure called prefix-growth with bounded gaps (*C-bounded-prefix-growth*). The procedure grows the prefix of a pattern and makes it longer by building and scanning its projected database under the rigid-length gap constraints.

#### Definition 5. (Projected database)

Let *Ψ *be a growing block, the projected database of *Ψ *is a complete collection of suffix of sequences *ξ*, where 〈*χξ*〉 is a suffix of a given sequence *S *∈ *D*, and *χ *∈ *S*/*Ψ*.

The technique of projected database is used recursively to project a sequence database into a smaller search space with respect to the growing pattern *Ψ *as prefix, and then the mining procedure scans the projected database with the consideration of gap constraints to count the support of symbols. A symbol is called a frequent symbol if the number of its occurrences satisfies the minimum support threshold. All of the locally frequent symbol *a *are appended to *Ψ *with a gap *i *to constitute a longer pattern: *Ψ *' = *Ψ*-x(*i*)-*a*.

**Figure 6:**
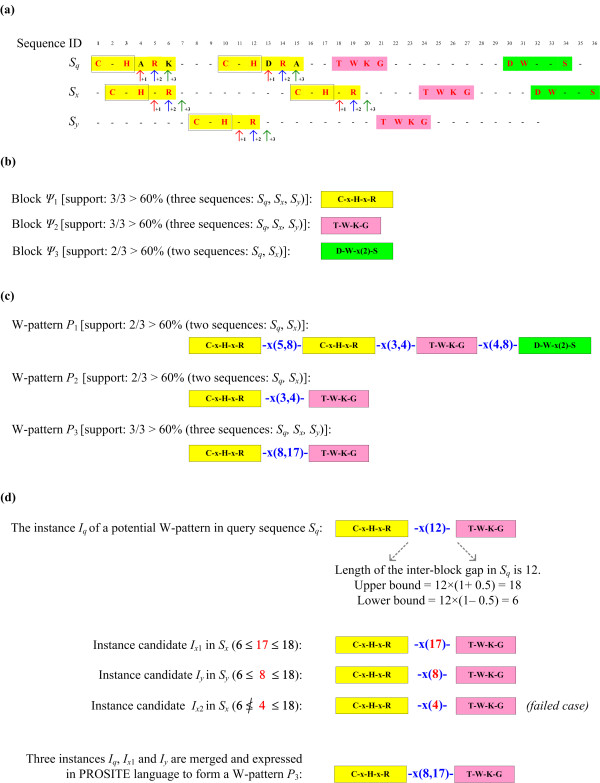
**A running example**. (a) A sample input sequence database (SDB) containing *S_q _*(reference sequence/query sequence), *S_x _*and *S_y_*. The solid arrows show the scanning range of the *bounded-prefix-growth *procedure under the intra-block gap constraint (*γ*_max _= 2) with respect to the pattern block 'C-x-H' (C - H) marked by black frames. The red, blue and green solid arrows denote the scanning residues with 'C-x-H' as the prefix and with gap lengths of zero, one, and two, respectively. Two other satisfied pattern blocks are marked by distinct background colours. The symbol '-' in the sequences represents the residues that cannot contribute to forming any patterns in this example; (b) Three satisfied pattern blocks; (c) Three satisfied W-patterns; (d) An example of how the maximum relative flexibility with respect to the reference sequence *S_q _*is employed to discover W-patterns.

The arguments of *C-bounded-prefix-growth *include a block *Ψ *and its projected database. Here, we present an example of scanning a projected database in Figure 6(a). This procedure takes a pattern *Ψ *= 'C-x-H' as input and tries to extend it under the user-specified intra-block gap constraints. In each call of *C-bounded-prefix-growth*, the search space of finding the next pattern symbol is bounded by the maximum length of an intra-block gap. A symbol in *Σ *is regarded as the candidate of the next symbol if the number of its occurrences in the projected database satisfies the minimum support threshold *λ *and its supporting sequences include the query protein *S_q_*. Each symbol is appended to the current pattern one at a time, and the resulting new block *Ψ*_1 _(in this example, *Ψ*_1 _= 'C-x-H-x-R') is used as the argument for the next call of *C*-*bounded-prefix-growth*, along with a possibly smaller projected database, because adding one more symbol to the current pattern reduces the size of the projected database. The process is recursively repeated until no satisfied symbol can be found in the current projected database.

##### Phase 2: growing long W-patterns

The second phase of the WildSpan aims to grow long patterns (W-patterns) that are composed of two or more blocks spanning large wildcard regions in protein sequences. Here we formally define what a W-pattern is.

#### Definition 6. (W-Pattern and inter-block gap)

A W-pattern *P *= *Ψ*_1_-x(*s*_1_,*e*_1_)-...- x(*s_p-1_*,*e_p-1_*)-*Ψ_p_*, where *Ψ*_1_, *Ψ*_2 _and *Ψ_p _*are the rigid-gapped blocks derived in phase 1. Any pair of adjacent blocks is connected by an inter-block gap, x(*s_i_*,*e_i_*) for *s_i _*≤ *e_i _*(1 ≤ *i *<*p*), which matches at least *s_i _*and at most *e_i _*arbitrary residues between blocks *Ψ_i _*and *Ψ_i+1_*. The flexibility of an inter-block gap x(*s_i_*,*e_i_*) is defined as *e_i _*- *s_i _*+ 1.

When the flexibility of an inter-block gap x(*s_i_*,*e_i_*) between two adjacent blocks is large, it implies that the pattern *P *spans a wildcard region and the length of the region is variant in its supporting sequences. It can be imagined that the mining results would be noisy if the growth of long patterns were not well confined. However, determining the extent of the flexibility of a wildcard region in advance is difficult. Fortunately, in the problem considered herein, this issue can be tackled by considering a relative flexibility constraint with respect to the length of the inter-block gap observed in the query sequence.

#### Definition 7. (Relative flexibility constraint of inter-block gaps)

A block *Ψ_i+1 _*is going to be appended to an existing W-pattern of which the last block is *Ψ_i_*. Let *l *be the length of an inter-block gap in the query sequence *S_q_*, connecting blocks *Ψ_i _*and *Ψ_i+1_*. The lower and upper bounds of this inter-block gap are defined as (1 - *f*_max_)×*l *and (1 + *f*_max_)×*l*, respectively, where *f*_max _is called the relative flexibility constraint. The resultant new W-pattern is satisfied if the number of supporting sequences that satisfies the lower and upper bounds of the inter-block gaps is equal to or exceeds the minimum support constraint *λ*.

Here we present an example of growing W-patterns when the constraint of relative flexibility is adopted. Let the minimum support threshold *λ *be 60%, the minimum length of block *κ*_min _be 3, the maximum intra-block gaps *γ*_max _be 2, the maximum relative flexibility *f*_max _be 50%, and the minimum number of blocks in a W-pattern *n*_min _be 2. Following the example used in Figure 6(a), the input sequence database has three satisfied blocks as shown in Figure 6(b): 'C-x-H-x-R', 'T-W-K-G', 'D-W-x(2)-S'. An advantage of specifying a query sequence in advance is that the repeats present in protein sequences can be properly dealt with. As presented in Figure 6(a), the two instances of block 'C-x-H-x-R' will be treated as two distinct block instances and they are distinguished by their starting positions, 1 and 10, in the query sequence *S_q_*. There are three satisfied W-patterns, as shown in Figure 6(c), according to the relative flexibility with respect to the query sequence *S_q_*. The first W-pattern has four blocks with supporting sequences *S_q _*and *S_x_*; and the second W-pattern has two blocks with supporting sequences *S_q_*, and *S_x_*; the third W-pattern has two blocks with supporting sequences *S_q_*, *S_x_*, and *S_y_*. Finally, the example of maximum relative flexibility for the third W-pattern is shown in Figure 6(d).

### Pruning strategy

A prefix-tree (or trie) is an ordered tree data structure for storing strings or sequences in a way that allows for fast pre-order traversal nodes. The branching factor is the number of descendants in the prefix tree. All the descendants of a node have a common prefix of the string associated with that node. As shown in Figure 6(a), the branching factor in each call of the *C-bounded-prefix-growth *procedure is bounded by one plus the maximum length of an intra-block gap, 1+*γ*_max_. On the other hand, the branching factor of *B-bounded-prefix-growth *that is invoked in the second phase depends to the number of blocks derived in the first stage, which is usually large. Aggressive pruning strategies are desired to achieve high efficiency of the proposed algorithm. These are described as below.

Both *C-bounded-prefix-growth *and *B-bounded-prefix-growth *grow patterns by searching the solution space in a depth-first manner. In this case, an algorithm must know when a branch can be pruned to reduce searching cost. The pruning strategies described in this subsection can be adjusted respectively for different phases of the WildSpan algorithm, in which different building components and constraints are considered. A node in a pattern tree of the solution space is promising if it corresponds to a substructure (a sub-pattern) of a valid solution (a pattern) without violating the user-specified constraints. To achieve high efficiency, a growing pattern should be pruned immediately as soon as it has been detected as a non-promising node.

The pruning of a node is based on exploiting the anti-monotonic property of this problem [34]. A constraint *C *is anti-monotonic if a sequence *β *that satisfies *C *has the property that every non-empty subsequence of *β *also satisfies *C*. The minimum support constraint serves as a good example to illustrate the anti-monotonic property. If a pattern *P *= *Ψ*_1_-x(*s*_1_,*e*_1_)-...-x(*s_p-1_*,*e*_*p*-1_)-*Ψ_p _*satisfies the minimum support constraint, then a sub-pattern that is composed of any subsets of the blocks in *P *also satisfies the minimum support constraint. Since our scanning procedure *B-bounded-block-growth *grows a W-pattern from a single block and tries to extend it by appending another block to it as the suffix of a new longer W-pattern, the anti-monotonic property can be exploited in the following way.

#### Pruning strategy 1

Let a pattern *P *be satisfied. If *P*' = *P*-x(*s_p_*', *e_p_*')-*Ψ_p_*' fails to be a satisfied pattern, then all of the patterns using *P*' as the prefix, *P*-x(*s_p_*', *e_p_*')-*Ψ_p_*'-x(*s_p_*", *e_p_*")-*Ψ_p_*" also fail to be satisfied. Thus, all the prefix-tree descendants nodes of *P*' can be pruned.

Pei *et al. *[34] proved that the minimum support constraint is anti-monotonic. The proof is straightforward, since a sub-pattern always matches more sequences in the database. Accordingly, this constraint works well with pruning strategy 1 in both phases of the WildSpan algorithm. The proposed relative flexibility constraint of inter-block gaps also has the anti-monotonic property.

**Theorem 1**. The relative flexibility constraint on the inter-block gaps is an anti-monotonic constraint. Given a pattern *P *= *Ψ*_1_-x(*s*_1_,*e*_1_)-...-x(*s*_*p*-1_,*e*_*p*-1_)-*Ψ_p_*, if *P *satisfies inter-block flexibility constraint, then so do all of its sub-patterns *P*' (*P*' can be obtained by deleting one or more blocks from pattern *P*.)

**Proof**: If *P *satisfies the relative flexibility constraint of inter-block gaps *f*_max_, it implies that every inter-block gap in *P *satisfies the same constraint. For a sub-pattern *P*', which is derived by deleting one block of length *c *from *P *in between two inter-block gaps of lengths *a *and *b *and of relative flexibilities *f_a _*and *f_b _*respectively, the maximum length of the resultant new inter-block gap equals to *a*×(1 + *f_a_*) + *b*×(1 + *f_b_*) + *c*, and the minimum length of the resultant inter-block gap equals *a*×(1 - *f_a_*) + *b*×(1 - *f_b_*) + *c*. Given that *f_a _*≤ *f*_max _and *f_b _*≤ *f*_max_, we have *a*×(1 + *f_a_*) + *b*×(1 + *f_b_*) + *c *≤ (*a *+ *b *+ *c*)×(1 + *f*_max_) and *a*×(1 - *f_a_*) + *b*×(1 - *f_b_*) + *c *≥ (*a *+ *b *+ *c*)×(1 - *f*_max_), which means the sub-pattern *P*' also satisfy the same constraint *f*_max_. This induction can be applied recursively if more than one block is deleted from *P *to form *P*'. Hence we can deduce that the relative flexibility constraint of inter-block gaps is an anti-monotone constraint.

The anti-monotonic property can be exploited more aggressively in the way described below.

#### Pruning strategy 2

Let a pattern *P *be satisfied. If *P*' = *P*-x(*s_p_*', *e_p_*')-*Ψ_p_*' fails to be a satisfied pattern, all patterns in the form of *P*-x(*s_p_*", *e_p_*")-*Ψ*_*p*_"-x(*s_p_*''', *e_p_*''')-*Ψ_p_*' also fail. Thus, for all other branches of the growing pattern *P*, for example, growing *P*-x(*s_p_*", *e_p_*")-*Ψ*_*p*_", *Ψ_p_*' is no longer a candidate block.

The upper bound constraint of a gap is not anti-monotonic, but it is prefix anti-monotonic [35]. A constraint *C_p _*is called prefix anti-monotonic if for a sequence *β *that satisfies *C_p_*, it implies that every prefix of *β *also satisfies *C_p _*[34]. Therefore, in the first phase, the procedure *C-bounded-prefix-growth *adopts a prefix-spanning mechanism, ensuring that the prefix of pattern *P *will be explored before pattern *P*. If a pattern does not satisfy the maximum gap constraint, then any pattern with that pattern as the prefix cannot satisfy the same constraint. Hence, pruning strategy 1 can be applied. Furthermore, when a query protein is involved during the mining process, it is regarded as one of the constraints. This constraint can also be easily proven to be anti-monotonic. In summary, pruning strategy 1 is adapted in both phases of WildSpan and pruning strategy 2 is applied only in the second phase.

### WildSpan algorithm

The WildSpan algorithm finds all the satisfied patterns with respect to a query sequence in two phases based the above strategies of search space pruning. In the first phase, WildSpan quickly mines all the closed blocks satisfying intra-block constraints with fixed-length gaps by using *C*-*bounded-prefix-growth *procedure, which constitute the building blocks of the W-patterns. After that, in the second phase, WildSpan discovers all the closed W-patterns satisfying inter-block constraints by connecting satisfied blocks found in the first phase with flexible gaps using *B-bounded-prefix-growth *procedure. The efficiency of WildSpan in finding W-patterns with large irregular gaps is ensured by exploiting the prefix anti-monotone characteristic of the new constraint model.

Based on the above algorithm description, we have the pseudo-code of WildSpan as shown in Figure A4.1 of Additional file 4, and two sub-procedures *C*-*bounded-prefix-growth *and *B*-*bounded-prefix-growth *are presented in Figure A4.2 and A4.3, respectively.

### Protein-based mining

The protein-based mining is designed for discovering protein functional regions of the query protein by referring to a set of its homologues. The default settings for W-patterns is: containing at least three blocks in one W-pattern and at least three conserved symbols in each block; requiring the length an intra-block gap is at most three, and the flexibility of an inter-block gap is no more than 50% with respect to the gap length observed on the query sequence. It is illustrated in Additional file 1 why this setting is suggested. WildSpan starts mining with the goal of finding the most highly supported W-patterns. For example, a support of 100% means that all the input set, including the query protein, satisfy the W-pattern. If such W-patterns do not exist, WildSpan decreases the setting gradually until at least one satisfied W-pattern has been found. All the results reported in this study are based the default settings, though the users can tighten or relax the constraints to improve the mining quality in different applications.

### Family-based mining

For applications of finding family signatures, the limitation of the proposed constraint model is that it might not be possible to find a satisfied W-pattern that matches all of the input sequences in a single run of protein-based mining. Hence, we proposed an iteratively mining strategy, family-based mining, for collecting a set of satisfied W-patterns that together serve as the diagnostic W-patterns for the input sequences. It is designed to proceed in the following manner: in the first run of WildSpan, the sequence of median length is selected from the input set as the query sequence. At the end of the first run, the W-pattern with the maximum support is picked. If not all of the input sequences match the selected W-pattern (such remaining sequences that do not match any of the selected W-patterns are called excluded sequences), the median-length sequence from the excluded sequences are assigned as the query sequence in the next call of WildSpan. In the second run, the W-pattern that matches the most excluded sequences of the first run will be picked. This procedure is repeated until the set of selected W-patterns covers all of the input sequences or no more W-patterns can be found from the remaining sequences.

## Availability and requirements

Project name: WildSpan

Project home page: http://biominer.csie.cyu.edu.tw/wildspan

Mirror site: http://biominer.bime.ntu.edu.tw/wildspan

Operating system(s): Linux

Programming language: C/C++

Other requirements: none

License: GNU GPL

## Competing interests

The authors declare that they have no competing interests.

## Authors' contributions

CMH designed and implemented the algorithm, performed all experiments and drafted the manuscript. CYC provided guidance on design of the methodology, participated in the discussion of biological significances and revised the manuscript. BJL aided in the guidance on the study and provided financial support. All authors read and approved the final manuscript.

## Supplementary Material

Additional file 1 **The analysis on the effect of changing parameter settings**. This file provides the analysis on the effect of changing parameter settings of WildSpan on the mining results. Click here for file

Additional file 2 **Experimental datasets and results for protein family classification**. This file provides the information of input datasets and complete results for the experiments of protein family classification. Click here for file

Additional file 3 **Closure checking schema**. This file provides the description of the closure checking schema employed by WildSpan to generate concise results. Click here for file

Additional file 4 **The complete pseudo codes for the WildSpan algorithm. **This file provides the complete pseudo codes for the WildSpan algorithm. Click here for file

## References

[B1] Livingstone CD Barton GJ Protein sequence alignments: a strategy for the hierarchical analysis of residue conservation Comput Appl Biosci 1993 9 6 745 756 8143162 10.1093/bioinformatics/9.6.745

[B2] Casari G Sander C Valencia A A method to predict functional residues in proteins Nat Struct Biol 1995 2 2 171 178 10.1038/nsb0295-171 7749921

[B3] Schueler-Furman O Baker D Conserved residue clustering and protein structure prediction Proteins 2003 52 2 225 235 10.1002/prot.10365 12833546

[B4] Califano A SPLASH: structural pattern localization analysis by sequential histograms Bioinformatics 2000 16 4 341 357 10.1093/bioinformatics/16.4.341 10869032

[B5] Neuwald AF Green P Detecting patterns in protein sequences J Mol Biol 1994 239 5 698 712 10.1006/jmbi.1994.1407 8014990

[B6] Rigoutsos I Floratos A Combinatorial pattern discovery in biological sequences: The TEIRESIAS algorithm Bioinformatics 1998 14 1 55 67 10.1093/bioinformatics/14.1.55 9520502

[B7] Wang JT Marr TG Shasha D Shapiro BA Chirn GW Discovering active motifs in sets of related protein sequences and using them for classification Nucleic Acids Res 1994 22 14 2769 2775 10.1093/nar/22.14.2769 8052532 PMC308246

[B8] Hsu CM Chen CY Liu BJ MAGIIC-PRO: detecting functional signatures by efficient discovery of long patterns in protein sequences Nucleic Acids Res 2006 34 Web Server issue W356 61 10.1093/nar/gkl309 16845025 PMC1538832

[B9] Hulo N Bairoch A Bulliard V Cerutti L De Castro E Langendijk-Genevaux PS Pagni M Sigrist CJ The PROSITE database Nucleic Acids Res 2006 34 Database issue D227 30 10.1093/nar/gkj063 16381852 PMC1347426

[B10] Wang J Han J Anonymous BIDE: Efficient Mining of Frequent Closed Sequences ICDE '04: Proceedings of the 20th International Conference on Data Engineering 2004 Washington, DC, USA: IEEE Computer Society 79

[B11] Wang K Xu Y Yu JX Anonymous Scalable sequential pattern mining for biological sequences Proceedings of the thirteenth ACM international conference on Information and knowledge management 2004 Washington, D.C., USA: ACM 178 187 full_text

[B12] Chakrabarti S Anand AP Bhardwaj N Pugalenthi G Sowdhamini R SCANMOT: searching for similar sequences using a simultaneous scan of multiple sequence motifs Nucleic Acids Res 2005 33 Web Server issue W274 6 10.1093/nar/gki493 15980468 PMC1160253

[B13] Keskin O Ma B Nussinov R Hot regions in protein--protein interactions: the organization and contribution of structurally conserved hot spot residues J Mol Biol 2005 345 5 1281 1294 10.1016/j.jmb.2004.10.077 15644221

[B14] Ogiwara A Uchiyama I Seto Y Kanehisa M Construction of a dictionary of sequence motifs that characterize groups of related proteins Protein Eng 1992 5 6 479 488 10.1093/protein/5.6.479 1438158 PMC7528547

[B15] Pisanti N Carvalho AM Marsan L Sagot M Lisbon I France IR Anonymous RISOTTO: Fast Extraction of Motifs with Mismatches Proceedings of the 7th Latin American Theoretical Informatics Symposium, 3887 of LNCS 2006 Valdivia, Chile: Springer-Verlag 757 768

[B16] Marsan L Sagot MF Algorithms for extracting structured motifs using a suffix tree with an application to promoter and regulatory site consensus identification J Comput Biol 2000 7 3-4 345 362 10.1089/106652700750050826 11108467

[B17] Carvalho AM Freitas AT Oliveira AL Rhône-alpes I Bernard UC I L Anonymous A highly scalable algorithm for the extraction of cis-regulatory regions Proceedings of the 3rd Asia Pacific Bioinformatics Conference, volume 1 of Advances in Bioinformatics and Computational Biology 2005 Imperial College Press 273 282 full_text

[B18] Klepper K Sandve GK Abul O Johansen J Drablos F Assessment of composite motif discovery methods BMC Bioinformatics 2008 9 123 10.1186/1471-2105-9-123 18302777 PMC2311304

[B19] Jonassen I Efficient discovery of conserved patterns using a pattern graph Comput Appl Biosci 1997 13 5 509 522 9367124 10.1093/bioinformatics/13.5.509

[B20] Saqi MA Sternberg MJ Identification of sequence motifs from a set of proteins with related function Protein Eng 1994 7 2 165 171 10.1093/protein/7.2.165 8170920

[B21] Blekas K Fotiadis DI Likas A Greedy mixture learning for multiple motif discovery in biological sequences Bioinformatics 2003 19 5 607 617 10.1093/bioinformatics/btg037 12651719

[B22] Frith MC Saunders NF Kobe B Bailey TL Discovering sequence motifs with arbitrary insertions and deletions PLoS Comput Biol 2008 4 4 e1000071 10.1371/journal.pcbi.1000071 18437229 PMC2323616

[B23] Narasimhan G Bu C Gao Y Wang X Xu N Mathee K Mining protein sequences for motifs J Comput Biol 2002 9 5 707 720 10.1089/106652702761034145 12487759

[B24] Hsu C Chen C Hsu C Liu B Efficient Discovery of Structural Motifs from Protein Sequences with Combination of Flexible Intra- and Inter-block Gap Constraints Advances in Knowledge Discovery and Data Mining 2006 530 539 full_text

[B25] Su CT Chen CY Hsu CM iPDA: integrated protein disorder analyzer Nucleic Acids Res 2007 35 Web Server issue W465 72 10.1093/nar/gkm353 17553839 PMC1933224

[B26] Chien TY Chang DT Chen CY Weng YZ Hsu CM E1DS: catalytic site prediction based on 1D signatures of concurrent conservation Nucleic Acids Res 2008 36 Web Server issue W291 6 10.1093/nar/gkn324 18524800 PMC2447799

[B27] Mintseris J Wiehe K Pierce B Anderson R Chen R Janin J Weng Z Protein-Protein Docking Benchmark 2.0: an update Proteins 2005 60 2 214 216 10.1002/prot.20560 15981264

[B28] Hsu CM Chen CY Liu BJ Huang CC Laio MH Lin CC Wu TL Identification of hot regions in protein-protein interactions by sequential pattern mining BMC Bioinformatics 2007 8 Suppl 5 S8 10.1186/1471-2105-8-S5-S8 17570867 PMC1892096

[B29] Altschul SF Madden TL Schaffer AA Zhang J Zhang Z Miller W Lipman DJ Gapped BLAST and PSI-BLAST: a new generation of protein database search programs Nucleic Acids Res 1997 25 17 3389 3402 10.1093/nar/25.17.3389 9254694 PMC146917

[B30] Bairoch A Apweiler R Wu CH Barker WC Boeckmann B Ferro S Gasteiger E Huang H Lopez R Magrane M Martin MJ Natale DA O'Donovan C Redaschi N Yeh LS The Universal Protein Resource (UniProt) Nucleic Acids Res 2005 33 Database issue D154 9 10.1093/nar/gki070 15608167 PMC540024

[B31] Chien TY Chang DT Chen CY Weng YZ Hsu CM E1DS: catalytic site prediction based on 1D signatures of concurrent conservation Nucleic Acids Res 2008 36 Web Server issue W291 6 10.1093/nar/gkn324 18524800 PMC2447799

[B32] Su CT Chen CY Hsu CM iPDA: integrated protein disorder analyzer Nucleic Acids Res 2007 35 Web Server issue W465 72 10.1093/nar/gkm353 17553839 PMC1933224

[B33] Hsu CM Chen CY Liu BJ Huang CC Laio MH Lin CC Wu TL Identification of hot regions in protein-protein interactions by sequential pattern mining BMC Bioinformatics 2007 8 Suppl 5 S8 10.1186/1471-2105-8-S5-S8 17570867 PMC1892096

[B34] Pei J Han J Wang W Anonymous Mining sequential patterns with constraints in large databases Proceedings of the eleventh international conference on Information and knowledge management 2002 McLean, Virginia, USA: ACM 18 25

[B35] Orlando S Perego R Silvestri C Anonymous A new algorithm for gap constrained sequence mining SAC '04: Proceedings of the 2004 ACM symposium on Applied computing. Nicosia, Cyprus edition 2004 New York, NY, USA: ACM 540 547

[B36] Lin M Lee S Wang S DELISP: Efficient Discovery of Generalized Sequential Patterns by Delimited Pattern-Growth Technology Advances in Knowledge Discovery and Data Mining 2002 198 209

