# Supplementary material for: WildSpan: mining structured motifs from protein sequences
Source: Algorithms Mol Biol. 2011 Mar 31;6:6. doi: 10.1186/1748-7188-6-6 (PMC3082213; doi:10.1186/1748-7188-6-6)
Supplement: Additional file 1 — The analysis on the effect of changing parameter settings. This file provides the analysis on the effect of changing parameter settings of WildSpan on the mining results. [file 1748-7188-6-6-S1.DOC]

**Additional File 1: supplemental data**

**WildSpan: mining structured motifs from protein sequences**

Chen-Ming Hsu^1^, Chien-Yu Chen^2,*^ and Baw-Jhiune Liu^3^

^1^Department of Computer Science and Information Engineering, Ching Yun University, Jung-Li, 320, Taiwan, R.O.C., ^2^Department of Bio-Industrial Mechatronics Engineering, National Taiwan University, Taipei, 106, Taiwan, R.O.C., and ^3^Department of Computer Science and Engineering, Yuan Ze University, Jung-Li, 320, Taiwan, R.O.C.

This supplement provides the analysis and suggestions on the parameter settings of WildSpan.

In this document, three important parameters were analyzed to investigate to their effects on the mining results of WildSpan: (1) the upper-bound of an intra-block gap: *γ* _max_; (2) the minimum size of a pattern block: *k*_min_; and (3) the maximum flexibility of an inter-block gap: *f*_max_. The study is based on a dataset of the family of oxidoreductase FAD/NAD(P)-binding proteins [InterPro entry: IPR001433], which comprises 295 proteins between each pair of which the overall sequence identity is 30%.

As shown in Figure A1.1(a), more hit-closed pattern blocks (the definition of ‘hit-closed’ can be found in Additional file 3) are generated by relaxing the constraint of pattern blocks, i.e., increasing the upper bound on an intra-block gap *γ*_max_. However, the number of support-closed W-patterns (the definition of ‘support-closed’ can be found in Additional file 3) with two or more blocks does not grow with the number of hit-closed pattern blocks, suggesting that a W-pattern that corresponds to a functional region typically comprises short motif blocks with limited substitutions, rather than longer pattern blocks. Longer pattern blocks may be biologically meaningful on their own, but do not necessarily help to grow a W-pattern.

Figure A1.1(b) reveals how the minimum size constraint on a pattern block influences the mining results. This constraint should be set as a small number because sometimes only few residues are conserved in a local region of protein sequences. However, setting *k*_min_ to two might generate too many W-patterns that a biologist could not reasonable explore thoroughly. Meanwhile, a pattern block with only two symbols raises the probability of random matches. Accordingly, we suggest that users can begin with a pilot search by setting *k*_min_ to three. After that, it might happen that some residue or two of the residues are conserved but cannot be found by WildSpan in its primitive results. In this case, the singly conserved residues can be easily identified using multiple sequence alignment on the matched segments of the supporting sequences. By this way, the derived patterns can be enhanced with multiple sequence alignment to have both singly conserved residues and conservative substitutions well considered.

Next, the effect of the relative flexibility constraint *f*_max_ of inter-block gaps is considered. As observed in Figure A1.1(c), when no constraint is applied, one protein sequence on average matches a pattern twice with different combinations of positions. We argue that a real functional motif seldom match a protein sequence twice with different positions. This phenomenon demonstrates that the mining results may contain some false positives. In other words, the quality of the derived patterns is suspected.

In summary, for the first run of WildSpan, the three parameters discussed above are suggested to set as follows: (1) the upper-bound of an intra-block gap, *γ* _max_ = 3; (2) the minimum size of a pattern block, *k*_min_ = 3; and (3) the maximum flexibility of an inter-block gap, *f*_max_ = 50%. For the other parameters of WildSpan, the minimum support threshold (*λ*) and the minimum number of blocks in a W-pattern (*n*_min_), the released package of WildSpan will automatically adjust the settings according to property of the input data. That is, *λ* is adjusted accordingly in order to find at least one satisfied W-patterns containing *n*_min_ blocks. The automated tuning procedures are illustrated in Figure A1.2.

**
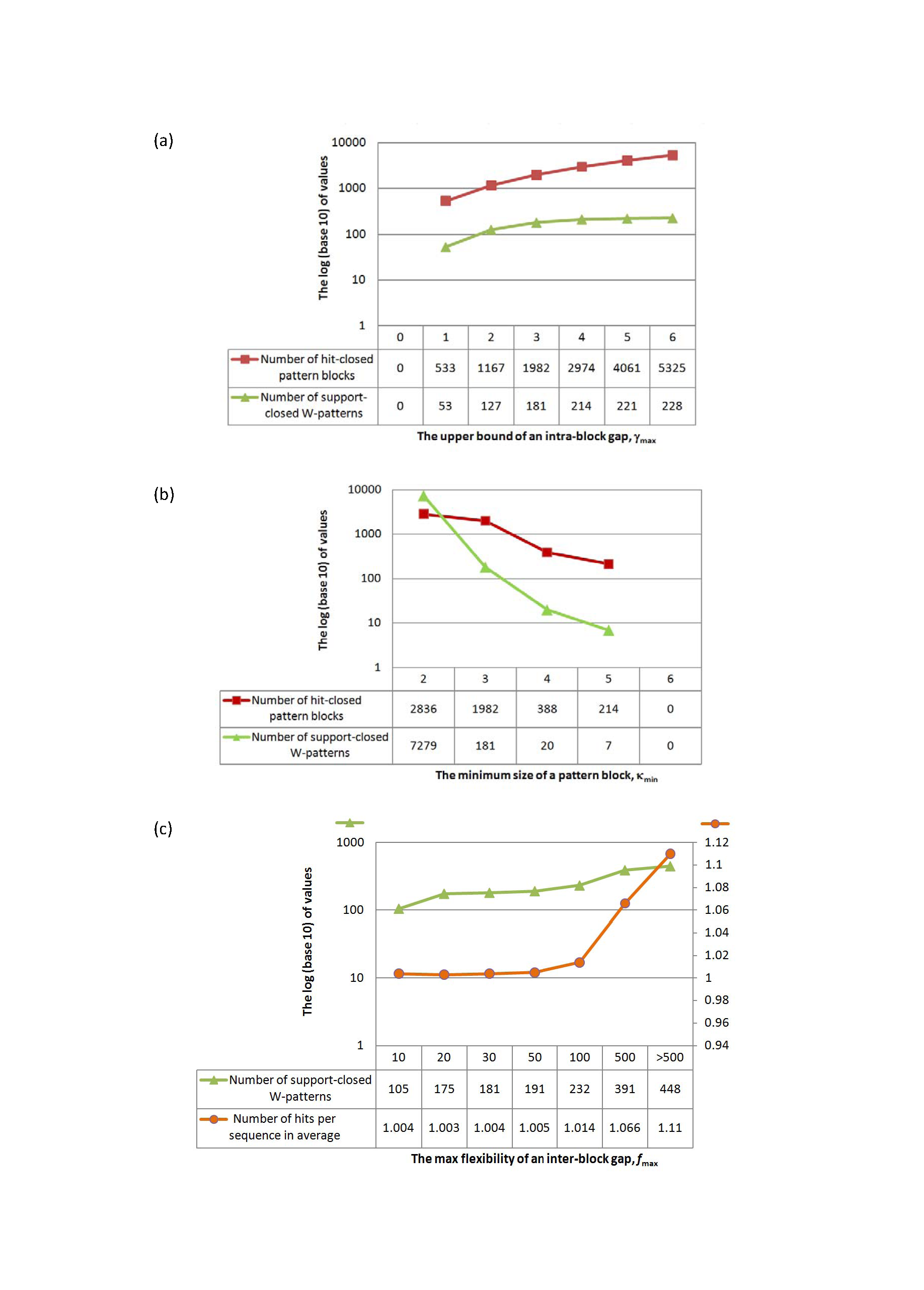
**

**Figure A1.1 – Study on effect of varying parameter settings.** Study on the effect of varying parameter settings based on the dataset of the Oxidoreductase FAD/NAD(P)-binding proteins (InterPro entry: IPR001433) with the minimum support constraint set as 15%. (a) The upper bound of an intra-block gap. (b) The minimum size of a pattern block. (c) The max flexibility of an inter-block gap.


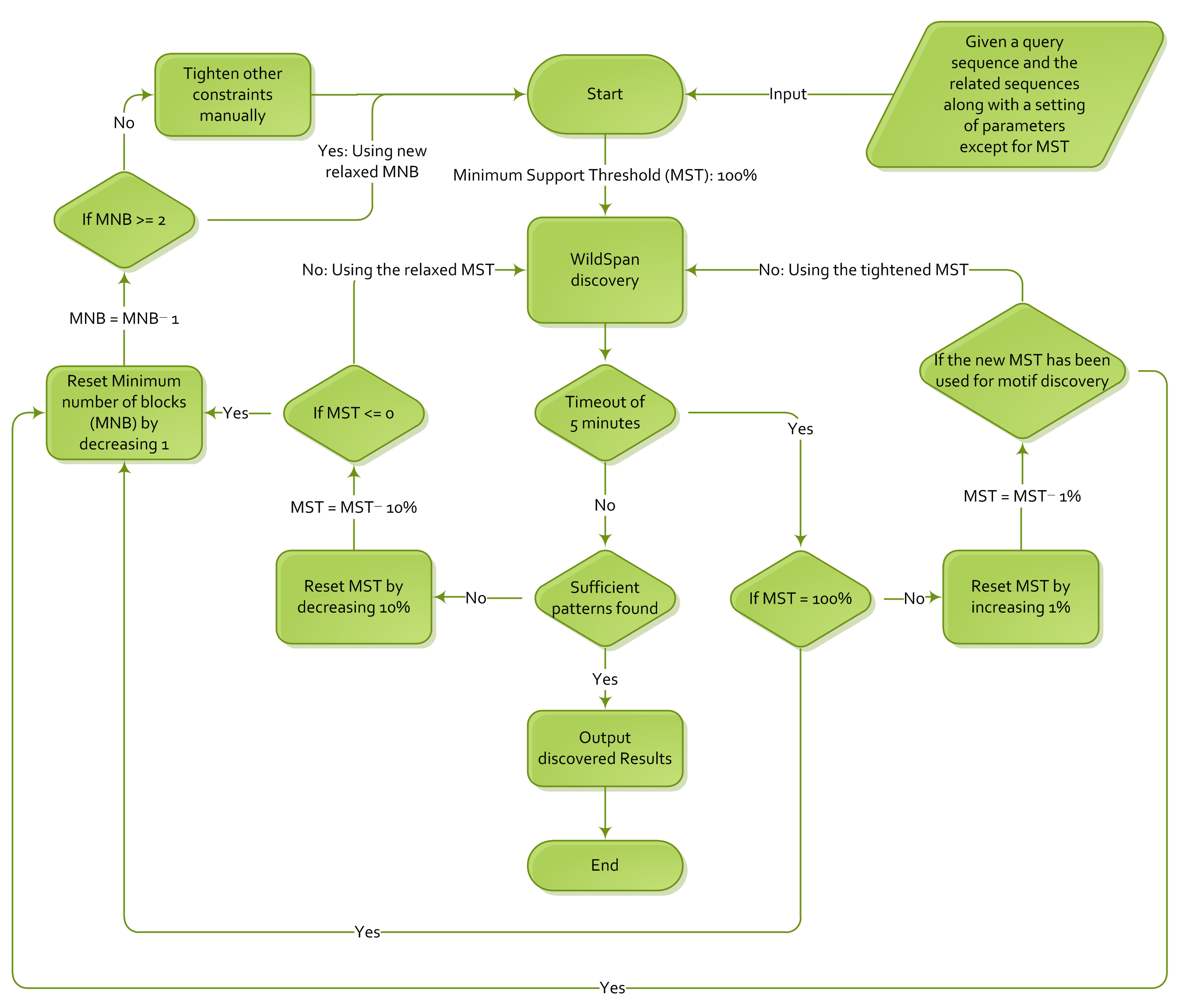


**Figure A1.2 – Automated parameter tuning of WildSpan.**
