# Supplementary material for: WildSpan: mining structured motifs from protein sequences
Source: Algorithms Mol Biol. 2011 Mar 31;6:6. doi: 10.1186/1748-7188-6-6 (PMC3082213; doi:10.1186/1748-7188-6-6)
Supplement: Additional file 2 — Experimental datasets and results for protein family classification. This file provides the information of input datasets and complete results for the experiments of protein family classification. [file 1748-7188-6-6-S2.DOC]

**Additional file 2: supplemental data**

**WildSpan: mining structured motifs from protein sequences**

Chen-Ming Hsu^1^, Chien-Yu Chen^2,*^ and Baw-Jhiune Liu^3^

^1^Department of Computer Science and Information Engineering, Ching Yun University, Jung-Li, 320, Taiwan, R.O.C., ^2^Department of Bio-Industrial Mechatronics Engineering, National Taiwan University, Taipei, 106, Taiwan, R.O.C., and ^3^Department of Computer Science and Engineering, Yuan Ze University, Jung-Li, 320, Taiwan, R.O.C.

This supplement provides the information of ten input datasets (PA10F) collected from the PROSITE database and the complete results for the experiments of protein family classification, as listed in Table A2.1 and A2.2 respectively. For each PROSITE entry, the proteins belonging to the target family were collected as the training data according to the cross-reference annotations provided in the release 50.9 of UniProtKB/Swiss-Prot (235673 entries). On the other hand, all the proteins in a newer version [Aug. 2010] of UniProtKB/Swiss-Port database (518415 entries) were collected as the testing data when conducting protein function classification for each PROSITE entry.

**Table A2.1 – Ten input datasets fed to different motif finding algorithms for generating sequence motifs for protein family classification (PA10F)**

| **PROSITE entry accession number** | **Description** | **Number of input sequences** | **Average sequence identity** | **Average sequence length** |
| --- | --- | --- | --- | --- |
| PS00301 | GTP-binding elongation factors signature | 1099 | 34% | 586 |
| PS00469 | Nucleoside diphosphate kinases active site | 320 | 48% | 167 |
| PS00455 | Putative AMP-binding domain signature | 296 | 26% | 973 |
| PS00111 | Phosphoglycerate kinase signature | 241 | 42% | 410 |
| PS00113 | Adenylate kinase signature | 235 | 39% | 218 |
| PS01071 | grpE Protein Signature | 201 | 32% | 203 |
| PS00627 | GHMP kinases putative ATP-binding domain | 196 | 20% | 341 |
| PS00387 | Inorganic pyrophosphatase signature | 120 | 38% | 208 |
| PS00112 | ATP:guanido phosphotransferases active site | 83 | 43% | 390 |
| PS00485 | Adenosine and AMP deaminase signature | 70 | 29% | 414 |
| Average | - | 296.1 | 35% | 391 |

* The training datasets were collected from the release 50.9 of UniProtKB/Swiss-Prot (235673 entries) according to the PROSITE cross-reference annotations.

**Table A2.2 – Experimental results for protein family classification**

| **Testing database**  Prediction results on the release 2010-08 of UniProtKB/Swiss-Port database (518415 entries) | | | | | | | | | | |
| --- | --- | --- | --- | --- | --- | --- | --- | --- | --- | --- |
| **Input dataset**  **PROSITE entry accession number** | **Method** | **Time used in seconds** | **True positive** | **True negative** | **False positive (FP) / Annotated by other database** | **False negative** | **Recall** | **Precision** | **Specificity** | **MCC** |
| **PS00301** | **PROSITE** | none | 2986 | 506824 | 0 / 0 | 136 | 95.64 | 100 | 100 | 0.978 |
|  | **RISOTTO** | 29.04 | 1584 | 506824 | 1 / 1 | 1538 | 50.737 | 99.937 | 100 | 0.711 |
|  | **Pratt** | 884 | 3052 | 506816 | 8 / 5 | 70 | 97.758 | 99.739 | 99.998 | 0.987 |
|  | **Teiresias** | 2.45 | 3121 | 165597 | 341227/NA | 1 | 99.968 | 0.906 | 62.673 | 0.054 |
|  | **WildSpan**  **(Family-based)** | 109.5 | 3120 | 506628 | 196 / 196 | 2 | 99.936 | 94.089 | 99.961 | 0.969 |
| **PS00469** | **PROSITE** | None | 591 | 509215 | 6 / 0 | 134 | 81.517 | 99.995 | 99.999 | 0.898 |
|  | **RISOTTO** | 2.68 | 398 | 509221 | 0 / 0 | 327 | 54.897 | 100 | 100 | 0.741 |
|  | **Pratt** | 12 | 660 | 509221 | 0 / 0 | 65 | 91.034 | 100 | 100 | 0.954 |
|  | **Teiresias** | 0.04 | 0 | 0 | 0 / 0 | 0 | 0 | 0 | 0 | 0 |
|  | **WildSpan (Family-based)** | 7.79 | 725 | 509220 | 1 / 1 | 0 | 100 | 99.862 | 100 | 0.999 |
|  | | | | | | | | | | |
| **Table A2.2 – Experimental results for protein family classification (continue)** | | | | | | | | | | |
| **PS00455** | **PROSITE** | None | 607 | 509261 | 23 / 0 | 55 | 91.692 | 96.349 | 99.995 | 0.94 |
|  | **RISOTTO** | 68.15 | 187 | 509284 | 0 / 0 | 475 | 28.248 | 100 | 100 | 0.531 |
|  | **Pratt** | 13773 | 387 | 509284 | 0 / 0 | 275 | 58.459 | 100 | 100 | 0.764 |
|  | **Teiresias** | 5.89 | 657 | 159224 | 350060/NA | 5 | 99.245 | 0.187 | 31.264 | 0.025 |
|  | **WildSpan (Family-based)** | 630.6 | 655 | 509169 | 115 / 6 | 7 | 98.943 | 85.065 | 99.977 | 0.917 |
| **PS00111** | **PROSITE** | None | 619 | 509241 | 0 / 0 | 86 | 87.801 | 100 | 100 | 0.937 |
|  | **RISOTTO** | 3.13 | 535 | 509239 | 2 / 1 | 170 | 75.887 | 99.628 | 100 | 0.869 |
|  | **Pratt** | 588 | 694 | 509231 | 10 / 1 | 11 | 98.440 | 98.580 | 99.998 | 0.985 |
|  | **Teiresias** | 0.23 | 697 | 246229 | 263012/NA | 8 | 98.865 | 0.264 | 48.352 | 0.036 |
|  | **WildSpan (Family-based)** | 10.06 | 705 | 509240 | 1 / 1 | 0 | 100 | 99.858 | 100 | 0.999 |
|  | | | | | | | | | | |
| **Table A2.2 – Experimental results for protein family classification (continue)** | | | | | | | | | | |
| **PS00113** | **PROSITE** | none | 801 | 509120 | 0 / 0 | 25 | 96.973 | 100 | 100 | 0.985 |
|  | **RISOTTO** | 4.28 | 588 | 509120 | 0 / 0 | 238 | 71.186 | 100 | 100 | 0.844 |
|  | **Pratt** | 9 | 764 | 509011 | 109 / 7 | 62 | 92.494 | 87.514 | 99.979 | 0.9 |
|  | **Teiresias** | 0 | 0 | 0 | 0 / 0 | 0 | 0 | 0 | 0 | 0 |
|  | **WildSpan (Family-based)** | 4.34 | 820 | 509116 | 4 / 2 | 6 | 99.274 | 99.515 | 99.999 | 0.994 |
| **PS01071** | **PROSITE** | none | 561 | 509354 | 2 / 0 | 29 | 95.085 | 99.645 | 100 | 0.973 |
|  | **RISOTTO** | 11.28 | 94 | 509356 | 0 / 0 | 496 | 15.932 | 100 | 100 | 0.399 |
|  | **Pratt** | 89 | 409 | 509356 | 0 / 0 | 181 | 69.322 | 100 | 100 | 0.832 |
|  | **Teiresias** | 0.08 | 568 | 128377 | 380979/NA | 22 | 96.271 | 0.149 | 25.204 | 0.021 |
|  | **WildSpan (Family-based)** | 63.5 | 585 | 509356 | 0 / 0 | 5 | 99.15 | 100 | 100 | 0.996 |
|  | | | | | | | | | | |
| **Table A2.2 – Experimental results for protein family classification (continue)** | | | | | | | | | | |
| **PS00627** | **PROSITE** | None | 480 | 509413 | 3 / 0 | 50 | 90.566 | 99.379 | 99.999 | 0.949 |
|  | **RISOTTO** | 64.71 | 44 | 509416 | 0 / 0 | 486 | 8.302 | 100 | 100 | 0.288 |
|  | **Pratt** | 588 | 327 | 509416 | 0 / 0 | 203 | 61.698 | 100 | 100 | 0.785 |
|  | **Teiresias** | 0.2 | 507 | 128376 | 381040/NA | 23 | 95.660 | 0.133 | 25.201 | 0.02 |
|  | **WildSpan (Family-based)** | 64.71 | 529 | 509399 | 17 / 4 | 1 | 99.811 | 96.866 | 99.997 | 0.983 |
| **PS00387** | **PROSITE** | None | 123 | 509713 | 102 / 0 | 8 | 93.893 | 54.667 | 99.980 | 0.716 |
|  | **RISOTTO** | 0.80 | 67 | 509815 | 0 / 0 | 64 | 51.145 | 100 | 100 | 0.715 |
|  | **Pratt** | 11 | 97 | 509815 | 0 / 0 | 34 | 74.046 | 100 | 100 | 0.86 |
|  | **Teiresias** | 0.04 | 131 | 478476 | 31339/NA | 0 | 100 | 0.416 | 93.853 | 0.063 |
|  | **WildSpan (Family-based)** | 1.55 | 130 | 509815 | 0 / 0 | 1 | 99.237 | 100 | 100 | 0.996 |
|  | | | | | | | | | | |
| **Table A2.2 – Experimental results for protein family classification (continue)** | | | | | | | | | | |
| **PS00112** | **PROSITE** | None | 108 | 509825 | 0 / 0 | 13 | 89.256 | 100 | 100 | 0.945 |
|  | **RISOTTO** | 1.19 | 54 | 509825 | 0 / 0 | 67 | 44.628 | 100 | 100 | 0.668 |
|  | **Pratt** | 4 | 111 | 509825 | 0 / 0 | 10 | 91.736 | 100 | 100 | 0.958 |
|  | **Teiresias** | 0.07 | 131 | 478476 | 31339/NA | 0 | 100 | 0.416 | 93.853 | 0.063 |
|  | **WildSpan (Family-based)** | 2.26 | 121 | 509825 | 0 / 0 | 0 | 100 | 100 | 100 | 1 |
| **PS00485** | **PROSITE** | None | 82 | 509690 | 20/0 | 154 | 34.75 | 80.39 | 99.99 | 0.528 |
|  | **RISOTTO** | 1.09 | 163 | 509710 | 0/0 | 73 | 69.068 | 100 | 100 | 0.831 |
|  | **Pratt** | 25 | 189 | 509560 | 150/0 | 47 | 80.085 | 55.752 | 99.971 | 0.668 |
|  | **Teiresias** | 0.08 | 184 | 159177 | 350533/NA | 52 | 77.966 | 0.052 | 31.229 | 0.018 |
|  | **WildSpan (Family-based)** | 3.51 | 222 | 509709 | 1/1 | 14 | 94.068 | 99.552 | 100 | 0.968 |
|  | | | | | | | | | | |
| **Table A2.2 – Experimental results for protein family classification (continue)** | | | | | | | | | | |
| **Average** | **PROSITE** | none | 695.8 | 509165.6 | 28.9/0 | 69 | 85.717 | 93.043 | 99.996 | 0.857 |
|  | **RISOTTO** | 18.635 | 371.4 | 509181 | 0.3/0.2 | 393.4 | 47.003 | 99.957 | 100 | 0.470 |
|  | **Pratt** | 1598.3 | 669 | 509154 | 27.7/1.3 | 95.8 | 81.507 | 94.159 | 99.995 | 0.815 |
|  | **Teiresias** | 0.908 | 599.6 | 194393 | 212953/NA | 11.1 | 76.798 | 0.2523 | 41.163 | 0.030 |
|  | **WildSpan (Family-based)** | 89.782 | 761.2 | 509148 | 33.5/23.6 | 3.6 | 99.042 | 97.481 | 99.993 | 0.990 |

NA: information not available because the number of false positives is too large to manually validate protein function.
