# Supplementary material for: WildSpan: mining structured motifs from protein sequences
Source: Algorithms Mol Biol. 2011 Mar 31;6:6. doi: 10.1186/1748-7188-6-6 (PMC3082213; doi:10.1186/1748-7188-6-6)
Supplement: Additional file 3 — Closure checking schema. This file provides the description of the closure checking schema employed by WildSpan to generate concise results. [file 1748-7188-6-6-S3.DOC]

**Additional file 3: supplemental data**

**WildSpan: mining structured motifs from protein sequences**

Chen-Ming Hsu^1^, Chien-Yu Chen^2,*^ and Baw-Jhiune Liu^3^

^1^Department of Computer Science and Information Engineering, Ching Yun University, Jung-Li, 320, Taiwan, R.O.C., ^2^Department of Bio-Industrial Mechatronics Engineering, National Taiwan University, Taipei, 106, Taiwan, R.O.C., and ^3^Department of Computer Science and Engineering, Yuan Ze University, Jung-Li, 320, Taiwan, R.O.C.

This supplement provides the description of closure checking schema used in WildSpan to generate concise results.

As the mining efficiency is concerned, it is desirable that the mining algorithms only generate closed patterns, i.e. a pattern having none of its super-patterns with the same occurrences (support) as it [1-4]. A closed pattern provides more succinct and complete information regarding a binding site or structural signature. It eliminates patterns that can be covered by any of its super-patterns with the same occurrences frequency during mining process. WildSpan employs a closure checking scheme in consideration of the constraint model (W-patterns) to generate concise results. It aggressively eliminates patterns that can be covered by any of its super-patterns with the same occurrences frequency during mining process.

First, we define the super-pattern as below.

Definition 1. (Super-pattern) Let a pattern *P* = *a*_1_-x(*i*_1_, *j*_1_)-*a*_2_-x(*i*_2_, *j*_2_)-…-x(*i_p_*_–1_, *j_p_*_–1_)-*a_p_*, the notation *P_m..n_*= *a_m_*-x(*i_m_*, *j_m_*)-*a_m_*_+1_-x(*i_m_*_+1_, *j_m_*_+1_)-…-x(*i_n_*_–1_, *j_n_*_–1_)-*a_n_* is used to denote the contiguous sub-pattern of *P*, which starts at the position of the *m*-th symbol and ends at the position of the *n*-th symbol of *P*, for 1 ≤ *m* ≤ *n* ≤ *p*. A pattern *P* is a super-pattern of *Q* if *Q* can be obtained by deleting pattern symbols *a_k_* (1 ≤ *k* ≤ *p*) from *P*, and in addition, one of following conditions holds: (1) *P*=*Q*-x(*i_p_*_–1_, *j_p_*_–1_)-*a_p_* (*a_p_* is the last symbol in *P*); (2) *P*=*a*_1_-x(*i*_1_, *j*_1_)-*Q* (*a*_1_ is the first symbol in *P*); (3) *k* (1 < *k* < *p*), *Q* = *P*_1.._*_k_*_–1_-x(*i_k_*_–1_+*i_k_*+1, *j_k_*_–1_+*j_k_*+1)-*P_k_*_+1.._*_p_*. 

Then we can define the concepts of support-closed and hit-closed.

Definition 2. (Closed pattern) A pattern *P* is support-closed if there is no super-pattern of *P* with the same number of supporting sequences in the input database. If there exists no super-pattern of *P* not only having the same support but also with the same appearing positions or anywhere else in the database, we call the pattern is hit-closed pattern. 

Support-closed is not effective as expected when growing the candidate blocks, because it ignores the all of the occurrences information of patterns. Therefore, we define another type of closure property that is more suitable for identifying blocks. The hit-closed closure checking scheme will be applied to the first phase of growing blocks, and support-closed closure checking scheme will be used in the pattern phase to reduce the number of W-patterns derived. The kernel method of closure checking is based on the CloSpan algorithm [4] with the additional consideration of gap constraints that we integrated.

**References**

1. Califano A: **SPLASH: structural pattern localization analysis by sequential histograms.** *Bioinformatics* 2000, **16**(4)**:**341-357.

2. Rigoutsos I, Floratos A: **Combinatorial pattern discovery in biological sequences: The TEIRESIAS algorithm.** *Bioinformatics* 1998, **14**(1)**:**55-67.

3. Wang J, Han J: **BIDE: Efficient Mining of Frequent Closed Sequences.** In *ICDE '04: Proceedings of the 20th International Conference on Data Engineering.* Edited by Anonymous Washington, DC, USA: IEEE Computer Society; 2004:79.

4. Yan X, Han J, Afshar R: **CloSpan: Mining Closed Sequential Patterns in Large Datasets.** In *In SDM.* Edited by Anonymous 2003:166-177.
