# Supplementary material for: WildSpan: mining structured motifs from protein sequences
Source: Algorithms Mol Biol. 2011 Mar 31;6:6. doi: 10.1186/1748-7188-6-6 (PMC3082213; doi:10.1186/1748-7188-6-6)
Supplement: Additional file 4 — The complete pseudo codes for the WildSpan algorithm. This file provides the complete pseudo codes for the WildSpan algorithm. [file 1748-7188-6-6-S4.DOC]

**Additional file 4: supplemental data**

**WildSpan: mining structured motifs from protein sequences**

Chen-Ming Hsu^1^, Chien-Yu Chen^2,*^ and Baw-Jhiune Liu^3^

^1^Department of Computer Science and Information Engineering, Ching Yun University, Jung-Li, 320, Taiwan, R.O.C., ^2^Department of Bio-Industrial Mechatronics Engineering, National Taiwan University, Taipei, 106, Taiwan, R.O.C., and ^3^Department of Computer Science and Engineering, Yuan Ze University, Jung-Li, 320, Taiwan, R.O.C.

This supplement provides the complete pseudo codes for the WildSpan algorithm.

The WildSpan algorithm (Figure A4.1) aims to discover W-patterns with a two-phase mining strategy. In the first phase, WildSpan invokes the Subroutine 1 (Figure A4.2) to obtain the complete set of hit-closed pattern blocks (the definition of ‘hit-closed’ can be found in Additional file 3). This subroutine grows pattern blocks satisfying both block and intra-block gap constraints by recursively calling the subroutine *C-bounded-prefix-growth*. After that, in the second phase, the Subroutine 2 (Figure A4.3) is used to discover the complete set of support-closed long patterns (the definition of ‘support-closed’ can be found in Additional file 3) containing two or more pattern blocks and satisfying the inter-block gap constraint by executing the procedure *B-bounded-prefix-growth* recursively to connect frequent pattern blocks found in the first phase with large irregular gaps. The supplementary notations and complete pseudo codes of WildSpan algorithm are as follows:

**Notation 1. (Sub-pattern)** A pattern *Q* is a sub-pattern of *P* if *Q* can be obtained by deleting one or more exact symbol(s) from *P*, denoted as Q⊂_SP_P.

**Notation 2. (Pattern match)** We say that a sequence *S* matches the pattern *P* if *S* contains a substring that can be derived from *P* by substituting each wildcard symbol ‘x’ by an arbitrary symbol from *Σ*, denoted as *P* ⊂_p_ *S*.

**Notation 3. (Pattern matched instance)** The set of distinct instances of pattern *P* in sequence *S* is denoted as *Instance*(*S*)*_P_*, and *Instance-Num*(*S*)*_P_* represents the number of instances in *Instance*(*S*)*_P_*.

**Notation 4. (Pattern hits)** The set *S*/*P* stands for all the substrings of *S* that match pattern *P*, and the total number of matched substring in *S*/*P* for *S*∈*D* is the number of all occurrences of pattern *P* in *D* denoted as *HIT*(*P*)*_D_*.

**Notation 5. (Pattern support)** The distinct input sequences *S* ∈ *D* such that *S* matches *P* under the constraints in *θ*, denoted as *SUP*(*P*)*_D_*.

**Notation 6. (Pattern block size)** The size of a block is defined as the number of exact symbols inside it, denoted as |*Ψ*|_A-NUM_.

**Notation 7. (Projected database)** The projected database of *Ψ* is denoted as *D*|*Ψ*.

**Notation 8. (Number of blocks in pattern)** Any pair of adjacent blocks is connected by an inter-block gap, x(*s_i_*,*e_i_*), which matches at least *s_i_* and at most *e_i_* arbitrary residues between blocks *Ψ_i_* and *Ψ_i_*_+1_, for 1 ≤ *i* < |*P|*_PB-NUM_, where |*P|*_PB-NUM_ is the number of blocks in *P*.

**Notation 9. (Size of W-pattern)** The size of a W-pattern is defined as the number of exact symbols inside it, denoted as |*P*|_A-NUM_.

| **Algorithm WildSpan(*D*, *PS*, *Sq*, *λ*, *γ*_max_, *k*_min_, *f*_max_, *n*_min_)**   |  | | --- | | **Input:** (1) A sequence database *D*, (2) a reference sequence *Sq*, (3) the minimum support threshold *λ*, (3) the maximum length of an intra-block gap *γ*_max_, (4) the minimum exact symbols in a block *k*_min_, (5) the maximum flexibility *f*_max_ of an inter-block gap with respect to the reference sequence, (6) the minimum number of blocks in a W-pattern *n*_min_.  **Output:** The set of support-closed W-patterns *PS* which satisfy the requirement of constraints, and match the reference sequence *Sq*.  **Global Functions:**  *SUP*(*P*)*_D_*: the notation denotes as the support of pattern *P* in sequence database *D*.  *HIT*(*P*)*_D_*: the notation denotes as the number of occurrences of pattern *P* in sequence database *D*.  |*P*|_PB-NUM_ : the notation denotes as the number of blocks in the W-pattern *P*.  |*P*|_A-NUM_ : the notation denotes as the number of non-wildcard symbols inside the block or W-pattern *P*.  *|BS|*_BLOCK-NUM_: the notation denotes as the number of blocks in the set *BS*,  *Instances*(*S_q_*)*_B_*: the notation denotes as all instances of block *B* in reference sequence *S_q_*.  *InstanceNum*(*S_q_*)*_B_*: the notation denotes as the number of instances of block *B* that match substrings at different positions in reference sequence *S_q_*.  *Hit-closure-checking*(*BS*)*_B_*: remove blocks *y* ∈ *BS* absorbed by *B* from *BS* such that *y* ⊂_SP_ *B* and *HIT*(*y*)*_D_* = *HIT*(*B*)*_D_*;  *Support-closure-checking*(*PS*)*_P_*: remove W-patterns *x* ∈ *PS* absorbed by *P* from *PS* such that *x* ⊂_SP_ *P* and *SUP*(*x*)*_D_* = *SUP*(*p*)*_D_*;  **1:** *BS* ← ∅; //the set of satisfied blocks  **2:** *PS* ← ∅; //the set of satisfied W-patterns  **3:** *C-bounded-prefix-growth*(∅, *D*, -1, *γ*_max_, *k*_min_, *n*_min_, *BS*, *PS*);  **4:** **if** *|BS|*_BLOCK-NUM_ < *n*_min_ **do**  **5:**  **return** W-patterns *P***;**  **6: done**  **7:** sorting the set of blocks *BS* in increasing order by starting matching position with respect to the reference sequence *Sq* to form *BS*_order_.  **8:** *B-bounded-prefix-growth*(∅, *D*, *BS*_order_, *PS*);  **9:** **return** W-patterns *PS* expressed in PROSITE language; | |  |   **Figure A4.1 – The main pseudo-code of WildSpan** |
| --- | --- | --- | --- |
| | **Subroutine 1** *C-bounded-prefix-growth*(*B*, *D|B*, *γ*, *γ*_max_, *k*_min_, *n*_min_, *BS*, *PS*) | | --- | | **Input:** (1) *B* is a growing block, (2) *D|B* is *B*-pseudo-projected database, (3) *γ* is a scanning rigid length of gap.  **Output:** The set of hit-closed blocks *BS* and the set of support-closed W-pattern *PS*.  **1:** **if** *B* = ∅ **do**  **2:** scan *D* once to find the set *A* of length-1 symbols *a* ∈ *A* such that the query protein *Sq* and at least *λ* of the support sequences contain *a*. The *a*-pseudo projected database *D*|*a* for  *a*∈*A* is also constructed at the same time.  **3:** **else if**  scan the symbols at *γ* + 1 position in each projected sequences of *D*|*B* with rigid gap length *γ* to find the set *A* of frequent symbols such that the query sequence *Sq* and at least *λ* supporting sequences contain the symbol. The 〈*B*-x(*γ*)-*a*〉-pseudo projected database *D*|*_B_*_-x(_*_γ_*)-*_a_* for  *a* ∈ *A* are also constructed simultaneously.  **4: done**  **5: for each** symbol *a* ∈ *A* **do**  **6:** **for each** fixed-length gap *γ*' ∈ {0,..,*γ*_max_} **do**  **7: if** *B* = ∅ **do**  **8:** *B*' = *a*;  **9: else**  **10:** *B*' ← *B*-x(*γ*)-*a*;  **11: done**  **12:** **call** *C-bounded-prefix-growth*(*B*', *D|B*', *γ*', *γ*_max_, *k*_min_, *n*_min_, *BS*, *PS*);  **13:** **done**  **14:** **if** |*B*|_A-NUM_ ≥ *k*_min_ **do**  **15:** **if** ! *y* ∈ *BS* such that *B* ⊂_SP_ *y* and *HIT*(*B*)*_D_* = *HIT*(*y*)*_D_* **do**  **16:** *Hit-closure-checking*(*BS*)*_B_*;  **17:** **for each** *b* ∈ *Instances*(*S_q_*)*_B_* **do** *BS* ← *BS* ∪ *b*;  ***//*** *the block B may has two or more matched instances in S_q_.*  **18:** **if** |*B*|_NUM_ ≥ (*k*_min_×*n*_min_) **do**  **19: if** ! *p*∈ *PS* such that *B* ⊂_SP_ *p* and *SUP*(*B*)*_D_* = *SUP*(*p*)*_D_* **do**  **20:** *Support-closure-checking*(*PS*)*_B_*;  **21:** *PS* ← *PS* ∪ *B*;  **22: done**  **23:** **done**  **24:** **done**  **25: done**  **26: done**  **27: return**; | |
| **Figure A4.2 – The pseudocode of sub-procedure *C-bounded-prefix-growth***   | **Subroutine 2** *B-bounded-prefix-growth*(*P*, *D*|*P*, *P-PPB, PS*) | | --- | | **Input:** (1) *P* is a current growing W-pattern, (2) *D*|*P* is a *P*-pseudo projected database, (3) *P*−*PPB* is *P*-projected blocks.  **Output:** The set of support-closed W-patterns *PS*.  **1**: **if** *P* = ∅ **do**  **2: for each** block *B_i_* ∈ *P-PPB* taken in increasing order *i* **do**  **//** construct the set of *B_i_*-projected blocks *B_i_PB* from *P*-*PPB* that excepts overlapping blocks with respect to *B_i_*.  **3:** *B_i_PB* ={*P-PPB*[*i*+1],..,*P-PPB*[|*P-PPB|*_BLOCK-NUM_]};  **4:** *B-bounded-prefix-growth*(*B_i_*, *D*|*B_i_*, *B_i_PB, PS*)  **5: done**  **6: else if**  **7:** *PPB'* ← ∅; *FB* ← ∅;  **8:** **for each** promising block *B* ∈ *P-PPB* **do**  **9:** append *B* to *P* to form a new candidate pattern *PB* ← 〈*PB*〉;  **10:** **for each** alignment *PB* ' of *PB* in the reference sequence *Sq* **do**  **11:** let *l_q_* be the length of inter-block gap of *PB*' connecting pattern *P* and follows block *B* in the reference sequence *Sq*;  **12:** let *l*_min_ ← *l_q_* × (1 – *f*_max_) be the minimum length of inter-block gap;  **13:** let *l*_max_ ← *l_q_* × (1 + *f*_max_) be the maximum length of inter-block gap;  **14:** count the support *SUP*(*B*)*D|P* for *B* in *D|P* such that the inter-block gap *l_s_* between *P* and *B* in sequences *s* ∈ *D* satisfy flexibility constraint *l*_min_ ≤ *l_s_* ≤ *l*_max_.  **15:** **if** the support *SUP*(*B*)*_D_*|*_P_* ≥ λ **do**  **16:** *FB* ← *FB* ∪*PB*'; *PPB'* ← *PPB'* ∪ *B*;  **17:** **done**  **18: done**  **19: done**  **20:** **if** |*P*|_PB-NUM_ ≥ *n*_min_ **do**  **21:** **if** ! *p*∈ *PS* such that *P* ⊂_SP_ *p* and *SUP*(*P*)*_D_* = *SUP*(*p*)*_D_* **do**  **22: *Support-closure-checking*(*PS*)*_P_*;**  **23:** *PS* ← *PS* ∪ *P;*  **24:** **done**  **25:** **done**  **26:** **for each** pattern *fb* ∈ *FB* **do**  **27:** construct the *fb*-projected blocks *fb*-*PPB* from *PPB'*.  **28:** **call** *B-bounded-prefix-growth*(*fb*, *D|fb*, *fb*-*PPB*, *PS*);  **29:** **done**  **30: done**  **31: return;** | |

**Figure A4.3 –The pseudocode of sub-procedure *B-bounded-prefix-growth***
